# Mentorship in endocrinology training: a cross-sectional study of the United States and Europe

**DOI:** 10.1016/j.eclinm.2025.103377

**Published:** 2025-07-22

**Authors:** David Toro-Tobon, Heather Billings, Anina F. Peersen, Elizabeth J. Atkinson, Antoan S. Sojat, Ljiljana V. Marina, Irina Bancos

**Affiliations:** a Division of Endocrinology, Diabetes, Metabolism and Nutrition, Mayo Clinic, 200 First Street SW, Rochester, MN 55902, USA; b Department of Education Administration, Mayo Clinic, Rochester, MN, USA; c Division of Clinical Trials and Biostatistics, Mayo Clinic, Rochester, MN, USA; d Clinic of Endocrinology, Diabetes and Metabolic Diseases, University Clinical Centre of Serbia, Belgrade, Serbia; e Faculty of Medicine, University of Belgrade, Belgrade, Serbia

**Keywords:** Mentorship, Endocrinology, Medical education, Burnout, Professional development, Regional differences

## Abstract

**Background:**

Mentorship is crucial for developing both scientific and professional competencies in medical training, yet its role in endocrinology training remains underexplored. We aimed to assess the prevalence of mentorship in endocrinology trainees, analyse demographic and training programme factors, and evaluate the impact of mentor characteristics on trainee outcomes.

**Methods:**

We conducted a cross-sectional study of endocrinology trainees in the United States and Europe (23 countries) between June 2023 to January 2024. Participants were recruited via professional societies, email lists, and social media. Those who had completed more than 7 years of training post-medical school or had missing data on duration of training were excluded. A structured online questionnaire was developed using validated mentorship competency tools and adapted for regional nuances to collect data on demographics, mentorship experiences, academic productivity, and well-being. The primary outcome assessed the prevalence of mentorship; secondary outcomes evaluated the associations between mentor characteristics and self-reported academic productivity, satisfaction within the mentorship relationship, and levels of burnout and stress. Univariable and multivariable logistic regression analyses were conducted.

**Findings:**

Between June 10, 2023, and January 1, 2024, 250 respondents (154 from the U.S. and 96 from Europe; 70.0% women, 64.4% White), 75.8% reported having a mentor. Significant regional differences emerged: U.S. trainees predominantly self-selected their mentors (69.7% vs. 23.1% in Europe) and reported less frequent interactions (monthly or less vs. more than weekly in Europe). Univariable analyses revealed that attributes such as active listening, inspirational guidance, and personalised career support were strongly linked to enhanced academic productivity, higher training satisfaction, and reduced burnout. In multivariable models, inspirational guidance was a significant predictor of academic productivity among U.S. trainees (94.4% vs. 35.3%, OR: 54.72, 95% CI: 4.7–2255.9), while in Europe, mentors facilitating strategic goal-meeting was associated with decreased burnout (77.8% vs. 40.0%, OR: 5.49, 95% CI: 1.1–33.7) and inspirational guidance with markedly improved mentorship satisfaction (90.7% vs. 28.6%, OR: 51.86, 95% CI: 2.2–1177.2).

**Interpretation:**

Though the design precludes causal inference, these findings underscore the universal benefits of mentorship in endocrinology training and highlight that targeted mentor competencies are key drivers of trainee success. Tailoring mentorship frameworks to regional training contexts may optimise academic productivity, training satisfaction, and overall well-being. Future longitudinal and qualitative studies are needed to clarify causal pathways and evaluate the effectiveness of tailored mentorship interventions.

**Funding:**

The 10.13039/100006108National Center for Advancing Translational Sciences (NCATS).

**Research in context**

**Evidence before this study**

Previous research in medical education has consistently highlighted the benefits of mentorship on academic productivity, career development, and reduced burnout. However, studies specifically addressing mentorship in endocrinology training remain limited, particularly regarding regional differences. We searched PubMed for studies in English from database inception to February 15, 2025, using the terms “mentorship”, “endocrinology” and their variations. The search yielded 351 results. Although some studies have evaluated mentorship in various specialties, none have done so in endocrinology and there has been limited comparison of mentorship practices and their outcomes between the United States and Europe.

**Added value of this study**

This study provides the first comprehensive, cross-sectional evaluation of mentorship among endocrinology trainees across the United States and Europe. By employing a structured online questionnaire based on validated mentorship competency tools and tailored for regional nuances, we examined mentor selection, interaction frequency, and key mentor attributes associated with academic productivity, mentorship satisfaction, and burnout. Our findings emphasise the universal value of mentorship in endocrinology training and reveal significant regional variations. In the United States, trainees typically self-select mentors, cultivating flexible, long-term partnerships that blend personalised career guidance with formalised research frameworks and holistic well-being support. These relationships correlate with higher mentorship satisfaction and lower burnout rates, driven by mentee-driven priorities and institutional commitments to diversity. Conversely, European trainees often participate in mentorship systems integrated into residency structures, prioritising consistent, goal-oriented interactions that balance collaborative productivity with self-directed skill development. Though challenged by sporadic mentor availability and less standardised infrastructure, these programmes excel in cultivating efficiency and clinical independence, with productivity tied to leaders who exemplify inspirational mentorship within hierarchical, autonomy-focused training cultures.

**Implications of all the available evidence**

Though generalisability is a limitation and the design precludes causal inference, these findings underscore that effective mentorship is universally beneficial but must be adapted to regional training contexts. Integrating best practices from both U.S. and European mentorship models may enhance academic productivity, mentorship satisfaction, and overall trainee well-being in endocrinology. Future longitudinal and qualitative studies should therefore aim to clarify causal pathways and evaluate the effectiveness of targeted mentorship strategies across different training environments.

## Introduction

Medical education is structured around a well-designed and highly regulated curriculum that focuses on developing trainees' scientific knowledge and core professional skills. However, many trainees lack sufficient guidance in navigating other important aspects of career development, such as research, networking, administrative tasks, skills enhancement, work-life balance, and career advancement.^1^^,^^2^ Mentorship, defined as a dynamic and reciprocal relationship between an experienced mentor and a less experienced trainee that fosters the growth of both parties,^3^ is an effective strategy to support trainees as they navigate their complex career paths.^4^, ^5^, ^6^ Although the goals of mentoring are universal across the different fields of medicine, the issues that mentors and mentees frequently confront may be uniquely challenging for each specialty in medicine.

Limited research has been conducted on mentorship in clinical subspecialty training. The prevalence of mentorship varies widely, ranging from as low as 19% in adolescent medicine to as high as 93% in primary care training.^1^ Studies from other specialties suggest that having a mentor is associated with greater training satisfaction, enhanced research productivity, career advancement, academic promotion, increased interest in pursuing an academic career, and a higher likelihood of securing grants.^1^^,^^4^^,^^6^, ^7^, ^8^, ^9^ Although the goals of mentoring are consistent across medical fields, each specialty can present unique complexities that might influence mentorship. Despite the recognised importance of mentorship, no prior studies have examined its status within endocrinology training. Additionally, while mentorship practices likely differ by region due to variations in healthcare systems and training structures, little research has explored these regional differences.

Therefore, this study aimed to assess the prevalence of mentorship in endocrinology trainees in the United States and Europe, analyse demographic and training programme factors that could influence mentor-mentee relationships, and evaluate the impact of mentor characteristics on trainee outcomes. Additionally, we sought to identify key factors that contribute to successful mentorship in endocrinology, provide insights into regional differences, and highlight areas for improvement in training programmes to enhance professional development for future endocrinologists.

## Methods

### Study design, population, and ethics

This cross-sectional study was conducted and reported in accordance with the Strengthening the Reporting of Observational studies in Epidemiology (STROBE) statement and took place among current endocrinology trainees in the United States and Europe between June 2023 and January 2024. In the United States, trainees were invited to participate through various channels, including emails distributed via the Endocrine Fellows Foundation, publicly available contact information of self-identified Fellows-in-Training members of the American Association of Clinical Endocrinologists (AACE), and publicly accessible contact details of programme coordinators and programme directors listed in the Fellowship and Residency Electronic Interactive Database Access (FREIDA). Invitations were also shared through social media posts on X (formerly Twitter) and endocrinology trainee texting groups. In Europe, trainees were recruited through the Society for Endocrinology, the European Society of Endocrinology’s Young Endocrinologists and Scientists (EYES), COST Action CA 20122 Harmonisation, the European Network for the Study of Adrenal Tumors (ENSAT), and social media posts on X (formerly Twitter). The invitation to participate included a link to an online questionnaire. Trainees who were willing to participate acknowledged their consent before proceeding to complete the questionnaire. Participants who had completed more than 7 years of training post medical school or did not provide a response to the question on duration of training were excluded.

The ethics of this study were reviewed and approved by the Education Research Committee and the Institutional Review Board at Mayo Clinic, Rochester, MN (22-010405). Written informed consent was obtained at the start of the online questionnaire, and all data were de-identified to ensure confidentiality. Participation was voluntary, with no compensation provided. The decision to participate was not disclosed to any evaluators and did not affect grading.

### Questionnaire

The online questionnaire was administered through Research Electronic Data Capture (REDCap) and designed based on previously identified mentorship characteristics, as well as previously validated tools for mentoring competency assessment,^1^^,^^10^ and included three parts: a) trainees’ demographic characteristics and measures of academic success, b) trainees’ current mentorship experience, and c) trainee’s perception on qualities of their mentors/mentorship team. There were two versions of the questionnaire: one for the United States and another for Europe. Both versions addressed the same variables, but the terminology was adjusted in some instances to account for regional differences. For the core sections assessing mentorship experiences, characteristics, and outcomes (derived from validated mentorship competency tools), both versions addressed the same fundamental variables; the primary adaptation to account for regional nuances in these sections consisted of adjusting terminology in some questions to ensure clarity, cultural appropriateness, and contextual relevance for respondents in each region. However, a small number of distinct questions related specifically to demographics and training programme characteristics (e.g., ‘Endocrinology training structure,’ ‘Duration of endocrinology training programme’) differed between the U.S. and European versions to accurately capture the inherently different training pathways.

### Outcomes

The primary outcome assessed the prevalence of mentorship, defined as “a dynamic, reciprocal relationship in a professional environment between an experienced career incumbent (mentor) and a novice (mentee), aimed at fostering the development of both”.^3^ Secondary outcomes evaluated the associations between mentor characteristics and self-reported academic productivity, satisfaction within the mentorship relationship, and levels of burnout and stress.

### Statistical analysis

Our prespecified analysis plan included descriptive statistics, group comparisons, and regression modeling. Continuous participant characteristic variables were reported as median and interquartile range (Q1, Q3) and quantitative variables were reported as count (%). Group differences were compared using the Kruskal–Wallis test for continuous variables and the chi-squared test for categorical. Prespecified univariable and multivariable logistic regression models were fit to better understand the relationship of mentoring characteristics with key study endpoints after adjusting for years of training after medical school and sex. The specific mentoring characteristics included as predictors in the multivariable logistic regression models were selected a priori based on their theoretical relevance to the study endpoints (academic productivity, mentorship satisfaction, and burnout) and their established importance in the mentorship literature.

As post-hoc analysis we also tested for effect modification by geographic region, additional multivariable models were fit including an interaction term between key mentor characteristics and region.

A two-tailed probability value of p < 0.05 was considered statistically significant for all tests. Statistical analysis was conducted using R version 4.4.1 and SAS version 9.4.

### Role of the funding source

Funding for this study was limited to a grant from The National Center for Advancing Translational Sciences (NCATS) that supported the use of the Research Electronic Data Capture (REDCap) survey tool. The funder had no role in the study design; in the collection, analysis, or interpretation of data; or in the writing of this report.

## Results

### Sample characteristics and measures of academic success

**Fig. 1:**
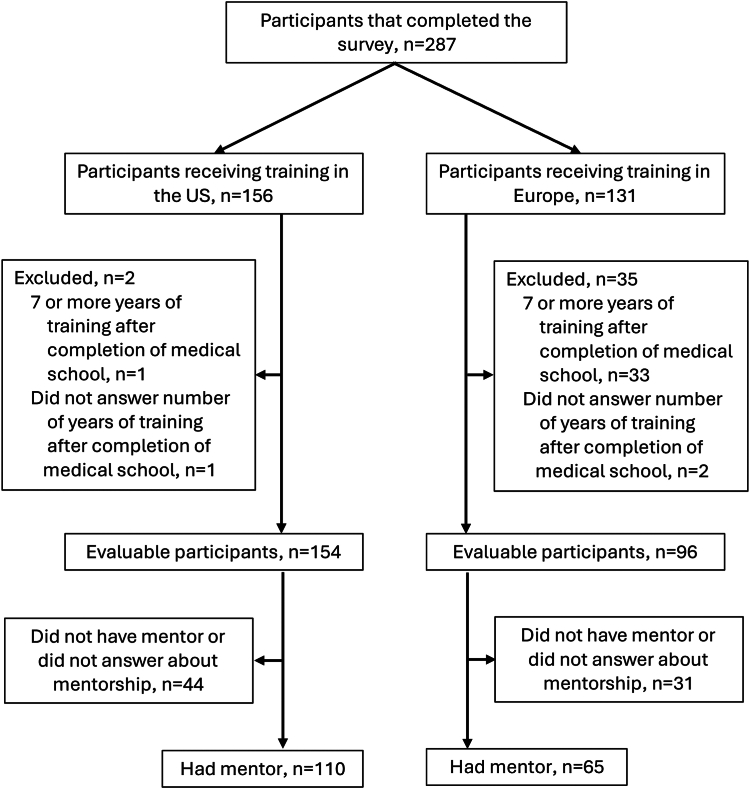
**Participant flowchart.** n: number of participants.

Between June 10, 2023, and January 1, 2024, a total of 287 participants completed the survey, including 156 from the United States (U.S.) and 131 from Europe (Fig. 1). Of these, 37 were excluded either because they reported seven or more years of training post-medical school, or they did not provide a years of training (2 U.S. survey and 35 Europe survey). Consequently, 250 participants were included in the statistical analysis, with 154 from the U.S. and 96 from Europe. The U.S. respondents represent approximately 22.3% of the estimated 691 endocrinology trainees the U.S., based on the average number of positions filled in the national residency matching programme for endocrinology in 2022 and 2023.^11^^,^^12^ For Europe, a comparable estimation of the total trainee pool was not feasible due to the significant heterogeneity in training programme structures and data reporting across different countries.

While 75.8% of participants reported having a mentor, with a predominance of women and a similar sex distribution across both regions (Table 1), regional disparities in race, training structures, research opportunities, and mentorship support were evident. Notably, in the U.S., the median age was 33.0 years (IQR 32.0–36.0), with 46.8% of trainees identifying as White, followed by 36.4% identifying as Asian. In contrast, in Europe, the mean age was 30.0 years (IQR 28.0–32.0), and most trainees were White (92.7%).

**Table 1:** Participant characteristics and access to mentorship.

	Europe (N = 96)	US (N = 154)	Total (N = 250)	p-value
**Country of training, n (%)**				N/A
Albania	1 (1.1%)	0 (0%)	1 (0.4%)	
Belarus	1 (1.1%)	0 (0%)	1 (0.4%)	
Belgium	1 (1.1%)	0 (0%)	1 (0.4%)	
Bosnia Herzegovina	1 (1.1%)	0 (0%)	1 (0.4%)	
Croatia	6 (6.3%)	0 (0%)	6 (2.4%)	
Denmark	2 (2.1%)	0 (0%)	2 (0.8%)	
France	6 (6.3%)	0 (0%)	6 (2.4%)	
Georgia	6 (6.3%)	0 (0%)	6 (2.4%)	
Germany	5 (5.3%)	0 (0%)	5 (2.0%)	
Greece	4 (4.2%)	0 (0%)	4 (1.6%)	
Italy	25 (26.3%)	0 (0%)	25 (10.0%)	
Netherlands	1 (1.1%)	0 (0%)	1 (0.4%)	
North Macedonia	1 (1.1%)	0 (0%)	1 (0.4%)	
Poland	2 (2.1%)	0 (0%)	2 (0.8%)	
Portugal	2 (2.1%)	0 (0%)	2 (0.8%)	
Romania	10 (10.5%)	0 (0%)	10 (4.0%)	
Serbia	7 (7.4%)	0 (0%)	7 (2.8%)	
Slovenia	1 (1.1%)	0 (0%)	1 (0.4%)	
Spain	1 (1.1%)	0 (0%)	1 (0.4%)	
Sweden	2 (2.1%)	0 (0%)	2 (0.8%)	
Turkey	3 (3.2%)	0 (0%)	3 (1.2%)	
Ukraine	3 (3.2%)	0 (0%)	3 (1.2%)	
United Kingdom	4 (4.2%)	0 (0%)	4 (1.6%)	
United States	0 (%)	154 (100%)	154 (61.8%)	
**Has a mentor**, n (%)				0.2420^a^
Yes	65 (80.2%)	110 (73.3%)	175 (75.8%)	
No	16 (19.8%)	40 (26.7%)	56 (24.2%)	
**Sex**, n (%)				0.5900^a^
Woman	66 (68.8%)	109 (70.8%)	175 (70.0%)	
Man	28 (29.2%)	44 (28.6%)	72 (28.8%)	
Prefer not to answer	2 (2.1%)	1 (0.6%)	3 (1.2%)	
**Race**, n (%)				<0.0001^a^
Asian	5 (5.2%)	56 (36.4%)	61 (24.4%)	
Black or African American	0 (0.0%)	6 (3.9%)	6 (2.4%)	
White	89 (92.7%)	72 (46.8%)	161 (64.4%)	
Prefer not to answer	2 (2.1%)	20 (13.0%)	22 (8.8%)	
**Age**,				<0.0001^b^
Median	30.0	33.0	32.0	
Q1, Q3	28.0, 32.0	32.0, 36.0	30.0, 35.0	
**Number of years in training after completion of medical school**, n (%)				<0.0001^a^
Less than 4	52 (54.2%)	140 (90.9%)	192 (76.8%)	
4	12 (12.5%)	14 (9.1%)	26 (10.4%)	
5	19 (19.8%)	0 (0.0%)	19 (7.6%)	
6	13 (13.5%)	0 (0.0%)	13 (5.2%)	
**Endocrinology training structure**^c^, n (%)				
Internal medicine residency followed by subspecialization in Endocrinology	37 (38.9%)	N/A	37 (38.9%)	
Residency in endocrinology and metabolic disease	55 (57.9%)	N/A	55 (57.9%)	
Other	3 (3.2%)	N/A	3 (3.2%)	
**Duration of endocrinology training program**^d^, n (%)				
2 years	N/A	103 (68.2%)	103 (68.2%)	
3 years	N/A	38 (25.2%)	38 (25.2%)	
More than 3 years	N/A	10 (6.6%)	10 (6.6%)	
**Number of endocrine trainees in program**				<0.0001^b^
Median	11.0	6.0	6.0	
Q1, Q3	6.0, 28.0	4.0, 8.0	4.0, 10.0	
**Research involvement**, n (%)				0.1439^a^
No	22 (22.9%)	35 (22.9%)	57 (22.9%)	
Yes–Clinical	53 (55.2%)	94 (61.4%)	147 (59.0%)	
Yes–Basic	9 (9.4%)	17 (11.1%)	26 (10.4%)	
Yes–Translational	12 (12.5%)	7 (4.6%)	19 (7.6%)	
**Percentage of training dedicated to research**				<0.0001^b^
Median	17.5	30.0	25.0	
Q1, Q3	5.0, 30.0	10.0, 50.0	10.0, 40.0	
**Number of publications**				0.2657^b^
Median	2.0	2.0	2.0	
Q1, Q3	0.0, 7.0	1.0, 4.0	1.0, 5.0	
**Number of abstracts, posters, and oral presentations**,				0.0044^b^
Median	6.0	4.0	5.0	
Q1, Q3	3.0, 14.0	2.0, 7.0	2.0, 8.5	
**Previously applied for a grant**, n (%)				0.0284^a^
Yes	35 (36.8%)	36 (23.8%)	71 (28.9%)	
No	60 (63.2%)	115 (76.2%)	175 (71.1%)	
**Academic appointment**, n (%)				0.0014^a^
None	74 (77.1%)	112 (73.7%)	186 (75.0%)	
Clinical instructor	3 (3.1%)	12 (7.9%)	15 (6.0%)	
Assistant professor	6 (6.3%)	24 (15.8%)	30 (12.1%)	
Associate professor	3 (3.1%)	0 (0.0%)	3 (1.2%)	
Other	10 (10.4%)	4 (2.6%)	14 (5.6%)	
**Satisfaction with endocrinology training**, n (%)				<0.0001^a^
Very satisfied	21 (21.9%)	62 (40.3%)	83 (33.2%)	
Satisfied	36 (37.5%)	70 (45.5%)	106 (42.4%)	
Neutral	24 (25.0%)	13 (8.4%)	37 (14.8%)	
Dissatisfied	13 (13.5%)	6 (3.9%)	19 (7.6%)	
Very dissatisfied	2 (2.1%)	3 (1.9%)	5 (2.0%)	
**Future type of practice intent**, n (%)				
Academic	61 (63.5%)	98 (63.6%)	159 (63.6%)	0.9879^a^
Private	35 (36.5%)	67 (43.5%)	102 (40.8%)	0.2701^a^
Industry	4 (4.2%)	13 (8.4%)	17 (6.8%)	0.1916^a^
Research	52 (54.2%)	21 (13.6%)	73 (29.2%)	<0.0001^a^
Clinical	82 (85.4%)	78 (50.6%)	160 (64.0%)	<0.0001^a^
Education scholar	15 (15.6%)	22 (14.3%)	37 (14.8%)	0.7718^a^
Other	1 (1.0%)	3 (1.9%)	4 (1.6%)	0.5786^a^
Unsure	2 (2.1%)	7 (4.5%)	9 (3.6%)	0.3095^a^
**Formal mentorship programs available at their institution**, n (%)				0.2663^a^
Yes	26 (32.9%)	61 (40.4%)	87 (37.8%)	
No	53 (67.1%)	90 (59.6%)	143 (62.2%)	
**Mentorship program available**, n (%)				
Mentorship committee	1 (1.0%)	17 (11.0%)	18 (7.2%)	0.0029^a^
IDP	9 (9.4%)	24 (15.6%)	33 (13.2%)	0.1583^a^
Standardized program	15 (15.6%)	27 (17.5%)	42 (16.8%)	0.6948^a^
Other	4 (4.2%)	8 (5.2%)	12 (4.8%)	0.7115^a^
**Barriers to finding a mentor**, n (%)				0.2622^a^
Yes	19 (23.5%)	26 (17.3%)	45 (19.5%)	
No	62 (76.5%)	124 (82.7%)	186 (80.5%)	
**Type of barriers encountered in finding a mentor**, n (%)				0.9992^a^
Limited mentorship opportunities	5 (41.7%)	10 (43.5%)	15 (42.9%)	
Insufficient mentor engagement	5 (41.7%)	9 (39.1%)	14 (40.0%)	
Personal barriers or mentee hesitation	1 (8.3%)	2 (8.7%)	3 (8.6%)	
Communication or cultural barriers	1 (8.3%)	2 (8.7%)	3 (8.6%)	
**Sufficient availability of faculty that could serve as mentors at their institution**, n (%)				0.0002^a^
Yes	52 (65.0%)	130 (86.1%)	182 (78.8%)	
No	28 (35.0%)	21 (13.9%)	49 (21.2%)	

Q1, Q3: First Quartile, Third Quartile; N/A: Not applicable.

a Chi-Square p-value.

b Kruskal–Wallis p-value.

c For U.S. respondents, this question was not applicable as all trainees follow a standardized pathway of internal medicine residency followed by subspecialization in endocrinology, as described in the text.

d For European respondents, this question was not applicable due to the greater heterogeneity and different overall structure of their training programs.

In Europe, most trainees (57.9%) were undergoing residency programmes dedicated to endocrinology and metabolic diseases, while all U.S. trainees followed a structured pathway of internal medicine residency followed by a subspecialisation in endocrinology. The duration of training also differed between regions, with European programmes being longer, as evidenced by a higher proportion of European trainees in training for five or more years after medical school (33.3% in Europe vs. 0% in the U.S., p < 0.0001). Additionally, European trainees reported larger cohort sizes, with a median of 11 trainees per programme, compared to 6 in the U.S. (p < 0.0001).

Research involvement varied between the regions. U.S. trainees dedicated a larger proportion of their training time to research (median percentage of training time: 30% vs. 17.5%, p < 0.0001), whereas European trainees were more engaged in translational research (12.5% vs. 4.6%, p = 0.1439). Additionally, European trainees were more likely to have applied for research grants (36.8% vs. 23.8%, p = 0.0284). While trainees from both regions had a similar median number of publications, European trainees had a higher median number of submitted abstracts and presentations (6.0 vs. 4.0, p = 0.0044).

Overall, only 37.8% of trainees reported having formal mentorship programmes at their institutions. When such programmes were available, they were predominantly either standardised programmes (16.8%) or individualised development plans (13.2%). U.S. trainees had higher access to mentorship committees compared to European trainees (11% vs. 1%, p = 0.0029). Despite this, barriers to finding a mentor were similar between the regions, with 19.5% of participants citing challenges such as limited mentorship opportunities and insufficient mentor engagement. However, European trainees reported significantly lower availability of faculty mentors who could serve in this role (65.0% vs. 86.1%, p = 0.0002).

There were no statistically significant differences in age, sex, race, or years of training after medical school between trainees with mentors and those without, in either the U.S. or Europe (Supplement eTable S1). However, trainees with mentors reported higher rates of satisfaction or neutrality with their endocrinology training in both regions (Europe: 90.8% vs. 71.0%, p = 0.0007; U.S.: 97.4% vs. 86.4%, p = 0.0072). In the U.S., trainees with mentors dedicated a higher percentage of time to research (median 30.0% vs. 15.0%, p = 0.0005), presented more abstracts, posters, and oral presentations (median 5.0 vs. 3.0, p = 0.0124), and were more likely to have applied for a grant (29.4% vs. 9.5%, p = 0.0104) compared to those without mentors. In the European cohort, only percentage of time dedicated to research different significantly between those with a mentor and those without (median 20.0% vs. 10.0%, p = 0.0346) (Supplement eTable S1).

### Mentorship characteristics

There were significant differences between mentees in Europe and the U.S. regarding the nature and impact of mentorship. U.S. mentees were more likely to select their mentor themselves (69.7% vs. 23.1%; p < 0.0001) and to interact with their mentors monthly or less (50.9% vs. 27.7%; p < 0.0001), whereas European mentees reported more frequent weekly or greater interactions (67.7% vs. 33.4%; p < 0.0001) (Table 2). Furthermore, there was a significant regional difference in the number of mentors reported by trainees. Overall, among mentored trainees who provided this information, 62.4% reported having two or more mentors. U.S. trainees were more likely to have multiple mentors, with 69.7% reporting two or more, compared to 50.0% of European trainees (p = 0.0154). Notably, while 89.2% of European mentees shared the same ethnicity as their mentor, only 22.9% of U.S. mentees did (p < 0.0001). However, the majority of both European and U.S. mentees did not consider it important for mentors and mentees to share the same ethnicity or gender identity (Table 2).

**Table 2:** Mentorship characteristics among participants reporting having a mentor.

	Europe (N = 65)	US (N = 110)	Total (N = 175)	p-value
**Number of mentors, n (%)**				
1	32 (50%)	33 (30.3%)	65 (37.6%)	0.0154^a^
2 or more	32 (50%)	76 (69.7%)	108 (62.4%)	
**Presence of a mentor outside of their training institution**, n (%)				0.3308^a^
Yes	17 (26.6%)	37 (33.6%)	54 (31.0%)	
No	47 (73.4%)	73 (66.4%)	120 (69.0%)	
**Source of outside mentor**, n (%)				
Medical school	6 (9.2%)	10 (9.1%)	16 (9.1%)	0.9753^a^
Another training program or institution	12 (18.5%)	20 (18.2%)	32 (18.3%)	0.9631^a^
Family related	1 (1.5%)	5 (4.5%)	6 (3.4%)	0.2908^a^
Community related	0 (0.0%)	3 (2.7%)	3 (1.7%)	0.1793^a^
Other	0 (0.0%)	9 (8.2%)	9 (5.1%)	0.0179^a^
**Mentor assignment**, n (%)				<0.0001^a^
Selected by the mentee	15 (23.1%)	76 (69.7%)	91 (52.3%)	
Selected by the training program leadership	43 (66.2%)	16 (14.7%)	59 (33.9%)	
Other	7 (10.8%)	17 (15.6%)	24 (13.8%)	
**Frequency of interaction with mentor**, n (%)				<0.0001^a^
Monthly or less	18 (27.7%)	55 (50.9%)	73 (42.2%)	
Bi-weekly	3 (4.6%)	17 (15.7%)	20 (11.6%)	
Weekly	14 (21.5%)	26 (24.1%)	40 (23.1%)	
More than weekly	30 (46.2%)	10 (9.3%)	40 (23.1%)	
**Mentor has their same gender identity**, n (%)				0.2640^a^
Yes	32 (49.2%)	67 (61.5%)	99 (56.9%)	
No	28 (43.1%)	37 (33.9%)	65 (37.4%)	
Unsure	5 (7.7%)	5 (4.6%)	10 (5.7%)	
**Considers that mentor and mentee should have the same gender identity**, n (%)				0.1537^a^
Yes	3 (4.7%)	12 (11.0%)	15 (8.7%)	
No	61 (95.3%)	97 (89.0%)	158 (91.3%)	
**Mentor has their same ethnicity**, n (%)				<0.0001^a^
Yes	58 (89.2%)	25 (22.9%)	83 (47.7%)	
No	6 (9.2%)	79 (72.5%)	85 (48.9%)	
Unsure	1 (1.5%)	5 (4.6%)	6 (3.4%)	
**Considers that mentor and mentee should have the same racial identity**, n (%)				0.6319^a^
Yes	2 (3.1%)	5 (4.5%)	7 (4.0%)	
No	63 (96.9%)	105 (95.5%)	168 (96.0%)	
**Has experienced increased academic productivity as a result of their mentorship relationship**, n (%)				0.5610^a^
Yes	52 (80.0%)	91 (83.5%)	143 (82.2%)	
No	13 (20.0%)	18 (16.5%)	31 (17.8%)	
**Has experienced less burnout and stress as a result of their mentorship relationship**, n (%)				0.0253^a^
Yes	28 (43.1%)	66 (60.6%)	94 (54.0%)	
No	37 (56.9%)	43 (39.4%)	80 (46.0%)	
**Would recommend working with their mentor/mentorship team**, n (%)				0.8532^a^
Yes	59 (90.8%)	98 (89.9%)	157 (90.2%)	
No	6 (9.2%)	11 (10.1%)	17 (9.8%)	
**Is satisfied by their mentorship relationship**, n (%)				0.1046^a^
Yes	49 (76.6%)	94 (86.2%)	143 (82.7%)	
No	15 (23.4%)	15 (13.8%)	30 (17.3%)	
**Has experienced negative outcomes as a result of their mentorship relationship**, n (%)				0.8358^a^
Yes	2 (3.1%)	4 (3.7%)	6 (3.4%)	
No	63 (96.9%)	105 (96.3%)	168 (96.6%)	
**Their mentor has used the mentorship relationship to advance their own career**, n (%)				0.2320^a^
Yes	17 (26.2%)	38 (34.9%)	55 (31.6%)	
No	48 (73.8%)	71 (65.1%)	119 (68.4%)	
**The mentorship relationship has helped them advance their career**, n (%)				0.6654^a^
Yes	57 (87.7%)	97 (89.8%)	154 (89.0%)	
No	8 (12.3%)	11 (10.2%)	19 (11.0%)	

a Chi-Square p-value.

Mentorship outcomes were largely positive across regions, with 82.2% reporting increased academic productivity and 89.0% experiencing career advancement. However, U.S. mentees were more likely than European mentees to report reduced burnout and stress due to mentorship (60.6% vs. 43.1%; p = 0.0253). Both groups overwhelmingly recommended their mentorship relationships (90.2%) and expressed satisfaction (82.7%).

### Mentor characteristics

Overall, most respondents perceived their mentors positively across attributes such as active listening (84.3%), providing helpful feedback (84.2%), and developing trust (84.3%). However, U.S.-based mentees consistently reported higher agreement on mentorship attributes, with statistically significant differences favoring U.S. mentors in accommodating communication styles (81.5% vs. 67.2%; p = 0.042), coordinating with other mentors (80.4% vs. 55.2%; p = 0.0018), and accounting for sex and cultural differences (74.8% vs. 39.7%; p < 0.0001). Mentees in the U.S. were also more likely to agree that their mentor assisted in achieving work-life balance (74.1% vs. 43.1%; p = 0.0003), developing personalised career paths (79.6% vs. 61.4%; p = 0.0399), and helping mentees network effectively (78.5% vs. 58.6%; p = 0.0237) (Supplement eTable S2).

**Fig. 2:**
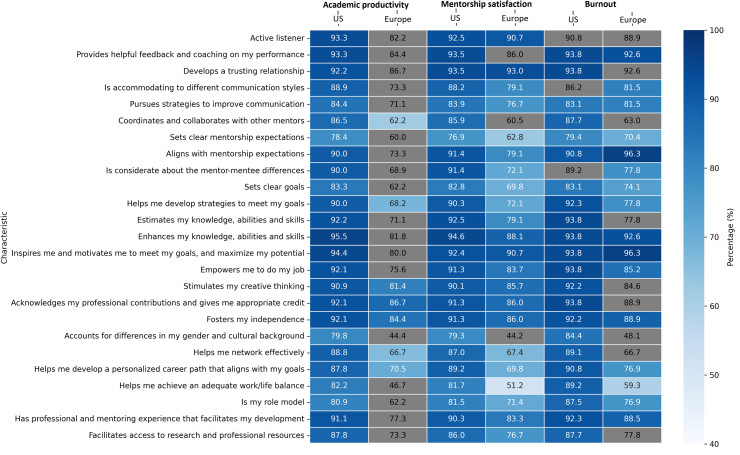
**Mentor attributes and their association with academic productivity, satisfaction, and burnout: a US–Europe Heatmap Analysis.** The heatmap depicts the association between mentorship characteristics and outcomes (academic productivity, mentorship satisfaction, and burnout) among trainees in the United States and Europe. The numerical annotation within each cell indicates the percentage of respondents endorsing that characteristic for the given outcome. Cells with statistically significant associations (p < 0.05) are color-coded, with the color intensityreflecting the percentage values. Cells representing non-significant associations (p > 0.05) are shown in gray. US: United States.

In the U.S. cohort, all evaluated mentor characteristics were positively correlated with increased academic productivity and mentorship satisfaction (Fig. 2, Supplement eTable S3). Among these, the strongest statistical associations (all p < 0.0001) were found with active listening, providing helpful feedback, developing trust, aligning with mentee expectations, fostering independence, setting clear goals, inspiring motivation, helping mentees develop personalised career paths, and enhancing mentees’ knowledge and skills. Mentees with lower levels of burnout and stress were more likely to have mentors who provided helpful feedback, developed trust, promoted career development, facilitated networking, aligned with expectations, and set clear goals (all p < 0.05). Additionally, fostering work-life balance, serving as role models, and empowering mentees were also associated with these lower burnout levels (all p < 0.001).

In the European cohort, several mentor characteristics were significantly associated with increased academic productivity among mentees (Fig. 2, Supplement eTable S4). The strongest associations included mentors acknowledging professional contributions and giving appropriate credit, helping mentees develop strategies to meet their goals, and fostering independence (all p-values < 0.01). Mentee satisfaction with their mentorship relationship was strongly associated with mentors who actively listened, developed a trusting relationship, accommodated diverse communication styles, helped mentees develop a path to meet their goals, inspired and motivated them to maximise their potential, stimulated creative thinking, and aligned with mentee expectations (all p-values < 0.001). Additionally, mentees who experienced less burnout and stress were significantly more likely to report that their mentors aligned with their expectations, inspired and motivated them to meet their goals, helped them develop strategies to meet their goals, and enhanced their knowledge, abilities, and skills (all p-values < 0.01).

### Univariable and multivariable analysis

**Fig. 3:**
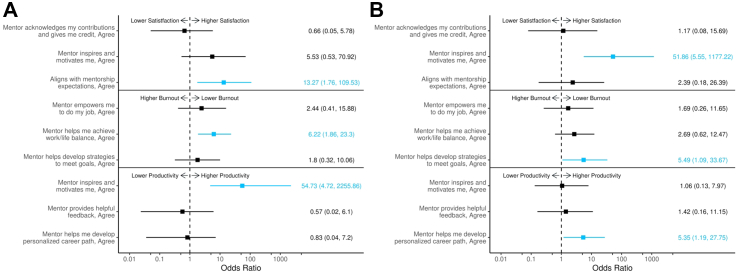
**Association between mentor characteristics and academic productivity, mentorship satisfaction, and burnout in the United States cohort. Panel A** corresponds to the U.S. cohort, and **Panel B** corresponds to the Europe cohort. The squares represent the odds ratios (ORs) and the horizontal error bars represent the 95% confidence intervals (CIs) from the multivariable logistic regression models. Data points are colored blue to indicate a statistically significant association (p < 0.05). The models shown are for: a) Increased productivity: adjusted for years of training after medical school, gender, mentor inspires and motivates, mentor provides helpful feedback, and mentor helps develop career path. b) Decreased burnout: adjusted for years of training after medical school, gender, mentor empowers, mentor helps achieve work/life balance, and mentor helps develop strategies to meet goals. c) Mentor satisfaction: adjusted for years of training after medical school, gender, mentor inspires and motivates, mentor acknowledges contributions and gives credit, and mentor aligns with expectations. OR: Odds Ratio; CI: Confidence Interval.

In the U.S. cohort, univariable logistic regression revealed that mentors who offered inspiration and motivation, provided constructive feedback, and assisted mentees in developing personalised career paths were significantly associated with increased academic productivity (OR: 30.80, p < 0.0001; OR: 13.83, p < 0.0001; and OR: 11.28, p < 0.0001, respectively). However, in multivariable analysis, only the association with mentors that provided inspiration and motivation remained significant (OR: 54.72, p = 0.0067; Table 3 and Fig. 3A). Mentors who supported work-life balance were significantly linked to decreased burnout (OR: 7.90, p < 0.0001), with this association persisting in multivariable analysis (OR: 6.21, p = 0.0040). Satisfaction with the mentorship relationship was most strongly associated with mentors who aligned with mentees expectations (OR: 38.95, p < 0.0001) and those who inspired and motivated mentees (OR: 21.85, p < 0.0001). However, only the first association remained significant in multivariable analysis (OR: 13.27, p = 0.0117). While the number of years of post-medical school training was positively associated with decreased burnout (OR: 2.01, p = 0.0319), it did not significantly predict academic productivity or mentorship satisfaction in multivariable analysis.

**Table 3:** Multivariable logistic regression analysis of a priori selected mentor characteristics associated with increased academic productivity, decreased burnout and satisfaction in the United States cohort.

Outcomes	Univariable analysis		Multivariable analysis	
	OR (95% CI)	p-value	OR (95% CI)	p-value
Increased academic productivity				
Number of years of training after medical school (per 1 year increase)	0.57 (0.308–1.052)	0.0711	1.10 (0.465–2.722)	0.8163
Sex (Men vs. Women)	1.06 (0.358–3.612)	0.9112	1.89 (0.430–10.538)	0.4245
Race (Non-White vs. White)	0.85 (0.301–2.361)	0.7618	–	–
Mentor helps me develop personalized career path (Agree vs. Disagree)	11.28 (3.714–37.049)	<0.0001	0.82 (0.036–7.195)	0.8807
Mentor provides helpful feedback (Agree vs. Disagree)	13.83 (4.116–50.788)	<0.0001	0.56 (0.024–6.104)	0.6630
Mentor inspires and motivates me (Agree vs. Disagree)	30.80 (8.582–130.147)	<0.0001	54.72 (4.725–2255.864)	0.0067
Decreased burnout				
Number of years of training after medical school (per 1 year increase)	1.07 (0.667–1.768)	0.7583	2.01 (1.087–3.966)	0.0319
Sex (Men vs. Women)	1.16 (0.505–2.774)	0.7211	2.19 (0.798–6.646)	0.1423
Race (Non-White vs. White)	0.79 (0.363–1.710)	0.5528	–	–
Mentor helps me achieve work/life balance (Agree vs. Disagree)	7.90 (3.074–22.559)	<0.0001	6.21 (1.865–23.304)	0.0040
Mentor helps develop strategies to meet goals (Agree vs. Disagree)	5.79 (2.008–19.367)	0.0020	1.80 (0.318–10.065)	0.4941
Mentor empowers me to do my job (Agree vs. Disagree)	7.24 (2.363–27.342)	0.0012	2.44 (0.406–15.881)	0.3258
Satisfaction with mentorship relationship				
Number of years of training after medical school (per 1 year increase)	0.48 (0.249–0.933)	0.0304	0.88 (0.329–2.406)	0.8057
Sex (Men vs. Women)	0.76 (0.241–2.672)	0.6540	1.38 (0.260–8.709)	0.7128
Race (Non-White vs. White)	1.77 (0.594–5.683)	0.3090	–	–
Mentor acknowledges my contributions and gives me credit (Agree vs. Disagree)	12.00 (3.505–43.754)	<0.0001	0.66 (0.050–5.776)	0.7255
Aligns with mentorship expectations (Agree vs. Disagree)	38.95 (9.972–201.791)	<0.0001	13.27 (1.763–109.531)	0.0117
Mentor inspires and motivates me (Agree vs. Disagree)	21.85 (6.021–90.608)	<0.0001	5.53 (0.527–70.925)	0.1629

All variables except race were included in multivariable analysis.

OR: Odds ratio; CI: Confidence interval.

In the European cohort, mentors who helped mentees develop a personalised career path were significantly associated with increased academic productivity in both univariable (OR: 5.36, p = 0.0143) and multivariable analyses (OR: 5.34, p = 0.0335) (Table 4, and Fig. 3B). Reduced burnout was significantly associated with mentors who facilitated goal-meeting strategies in both univariable and multivariable models (OR: 5.25, p = 0.0053; and OR: 5.490, p = 0.0470, respectively), while the number of years in training after medical school was significant only in multivariable analysis (OR 1.60, p = 0.0324). Satisfaction with mentorship was strongly associated with mentors who inspired and motivated mentees (univariable OR: 24.37, p < 0.0001; multivariable OR: 51.86, p = 0.0025).

**Table 4:** Multivariable logistic regression analysis of a priori selected mentor characteristics associated with increased academic productivity, decreased burnout and satisfaction in the European cohort.

Outcomes	Univariable analysis		Multivariable analysis	
	OR (95% CI)	p-value	OR (95% CI)	p-value
Increased academic productivity				
Number of years of training after medical school (per 1 year increase)	1.01 (0.711–1.463)	0.9366	1.05 (0.698–1.635)	0.7880
Sex (Men vs. Women)	0.39 (0.109–1.358)	0.1388	0.44 (0.106–1.721)	0.2401
Race (Non-White vs. White)	0.48 (0.042–10.829)	0.5622	–	–
Mentor helps me develop personalized career path (Agree vs. Disagree)	5.36 (1.473–22.844)	0.0143	5.34 (1.186–27.747)	0.0335
Mentor provides helpful feedback (Agree vs. Disagree)	2.41 (0.539–9.960)	0.2265	1.41 (0.161–11.145)	0.7410
Mentor inspires and motivates me (Agree vs. Disagree)	2.50 (0.630–9.523)	0.1785	1.06 (0.131–7.967)	0.9531
Decreased burnout				
Number of years of training after medical school (per 1 year increase)	1.16 (0.874–1.566)	0.3028	1.60 (1.073–2.591)	0.0324
Sex (Men vs. Women)	0.45 (0.149–1.318)	0.1576	0.33 (0.082–1.199)	0.1011
Race, Non-White	0.64 (0.029–7.111)	0.7289	–	–
Mentor helps me achieve work/life balance (Agree vs. Disagree)	3.55 (1.222–10.996)	0.0227	2.69 (0.619–12.472)	0.1883
Mentor helps develop strategies to meet goals (Agree vs. Disagree)	5.25 (1.709–17.979)	0.0053	5.49 (1.086–33.674)	0.0470
Mentor empowers me to do my job (Agree vs. Disagree)	3.63 (1.069–14.711)	0.0491	1.69 (0.261–11.655)	0.5774
Satisfaction with mentorship relationship				
Number of years of training after medical school (per 1 year increase)	0.76 (0.528–1.071)	0.1247	0.50 (0.209–0.910)	0.0528
Sex (Men vs. Women)	1.20 (0.363–4.366)	0.7700	1.25 (0.214–8.546)	0.8015
Race (Non-White vs. White)	0.59 (0.053–13.377)	0.6815	–	–
Mentor acknowledges my contributions and gives me credit (Agree vs. Disagree)	6.16 (1.615–25.280)	0.0086	1.16 (0.079–15.690)	0.9066
Aligns with mentorship expectations (Agree vs. Disagree)	9.44 (2.549–41.590)	0.0013	2.39 (0.177–26.386)	0.4756
Mentor inspires and motivates me (Agree vs. Disagree)	24.37 (5.688–132.394)	<0.0001	51.86 (5.554–1177.220)	0.0025

All variables except race were included in multivariable analysis.

OR: Odds ratio; CI: Confidence interval.

To formally test whether the effect of key mentor characteristics on outcomes differed by geographic region, we conducted further multivariable analyses incorporating interaction terms (Supplement eTable S5a–c). A statistically significant interaction was identified between a mentor providing inspiration and motivation and geographic region for the outcome of increased academic productivity (p = 0.042). This finding indicates that the magnitude of the association between having an inspirational mentor and academic productivity is statistically different in the U.S. compared to Europe. In contrast, for the outcomes of decreased burnout and increased mentorship satisfaction, the tested interaction terms between mentor characteristics and region were not statistically significant.

## Discussion

This is the first study to provide a comprehensive evaluation of mentorship in endocrinology training across two distinct regions—the US and Europe—and highlights both commonalities and key differences in mentorship prevalence, structure, and outcomes. Overall, 75.8% of trainees reported having a mentor, aligning with rates in other specialties (66–93%),^1^^,^^13^, ^14^, ^15^, ^16^ and underscoring the recognised importance of mentorship in fostering academic and professional development.^4^^,^^5^^,^^17^^,^^18^ However, our findings reveal significant regional variations in mentorship practices that appear to be influenced by differences in training pathways, programme structures, and cultural norms. Conversely, the findings from our study that trainees without mentors reported lower training satisfaction and, particularly in the U.S., demonstrated lower engagement in certain research activities, may suggest they face additional hurdles in perceived academic progression and goal attainment. This further underscores the critical role of establishing effective mentorship opportunities for all trainees.

Unlike other studies in male-dominated specialties,^1^^,^^19^ this study found no significant sex differences in mentorship access or academic outcomes. However, the stark contrast in racial homogeneity—92.7% White among European trainees vs. a more diverse U.S. cohort—highlights differences in ethnic representation that may influence access to culturally concordant mentorship. It is important to note, however, that Europe is not monolithic in this regard; the degree of racial and ethnic homogeneity varies considerably across different European countries, with some nations being historically more homogeneous while others feature more diverse populations. Our aggregate European data reflects the overall composition of our respondent sample from various European nations but may not fully capture the specific demographic makeup of every individual country included. As expected, racial congruence in mentorship was far more common in Europe than in the U.S. While prior studies suggest that trainees may prefer mentors of the same ethnicity or gender, the broader literature on mentorship and diversity suggests that ethnic concordance can offer distinct benefits for some mentees, particularly those from underrepresented racial and ethnic groups. These benefits may include enhanced feelings of validation, psychological safety, improved communication, and a deeper understanding of shared experiences or specific career navigation challenges within certain cultural contexts.^20^, ^21^, ^22^

The practical ramifications of our findings are nuanced. In the U.S., the relatively low rate of ethnic concordance within a diverse trainee population highlights the paramount importance of all mentors developing and demonstrating strong cultural competency skills. Our finding that U.S. mentees rated their mentors significantly higher on ‘accounting for differences in my sex and cultural background’ suggests that this skill is present and valued, potentially mitigating challenges arising from a lack of ethnic concordance for some. However, it also raises considerations about the systemic availability and visibility of ethnically diverse mentors in endocrinology. In Europe, the current high level of ethnic concordance is largely a reflection of the greater ethnic homogeneity within the trainee and mentor pool. As European countries become more diverse, training programmes may need to proactively develop strategies to support future minority trainees and foster culturally responsive mentorship, perhaps by learning from models or practices in more diverse settings. This points to the ongoing need to cultivate culturally responsive mentorship across all regions to ensure equitable support for an increasingly diverse trainee population.

Regional disparities in mentorship structures reflect divergent training pathways. U.S. trainees typically pursue endocrinology after completing a 3-year internal medicine residency followed by a 2–3 year fellowship. In contrast, the training pathways in Europe show more variation. While 57.9% of European trainees in our study reported being in residency programmes specifically dedicated to endocrinology and metabolic diseases, the routes for the remaining trainees reflect Europe's diverse national training systems and curriculum recommendations. Many European countries, in line with guidelines such as the European Society of Endocrinology (ESE) Curriculum and Training Recommendation, structure their pathways to include a foundational period of 1–3 years in General Internal Medicine followed by 3–5 years of specialised endocrinology training.^23^ Consequently, a significant portion of the remaining 42.1% of European trainees may be within this initial general medicine phase of their endocrinology track, or in broader internal medicine programmes with a clear intention and pathway to subspecialise in endocrinology. The typical configuration of these European pathways—often comprising a total of 6 or more years dedicated to general medicine and subsequent specialised endocrinology training—generally results in an overall training duration for endocrinologists that is longer than the common U.S. model of 5–6 years post-medical school. Moreover, European endocrinology training programmes, including those specifically dedicated to the specialty, generally accommodate larger trainee cohorts.

These structural differences may explain variations in mentorship dynamics, such as U.S. trainees more frequently self-selecting mentors but engaging less often (monthly vs. weekly in Europe), contrasting with Europe’s structured, residency-embedded programmes that foster regular interactions. These structural differences may also contribute to variations in mentorship engagement, such as the number of mentors trainees utilise. For instance, the higher prevalence of U.S. trainees reporting multiple mentors (69.7% vs. 50.0% in Europe) could reflect the tendency in the U.S. system to self-select diverse mentoring support to build a personalised career guidance network, a practice potentially less common in Europe’s more structured, residency-embedded programmes.

Divergent programme emphases also extended to research activities. U.S. trainees dedicated more time to research, whereas European trainees prioritised translational projects, grant applications, and conference presentations, reflecting divergent programme emphases. U.S. pathways, following internal medicine residency, emphasise structured research blocks, while Europe’s integrated, longitudinal training fosters clinical-academic immersion and efficiency in scholarly output. While median total publications were similar between U.S. and European trainees, European trainees reported significantly more submitted abstracts and presentations. Although both are valuable scholarly contributions, these metrics often carry different weights in academic evaluations; peer-reviewed publications, for instance, are frequently considered more critical for career advancement than abstracts. The higher volume of abstracts and presentations from European trainees could suggest varying regional emphases in training, such as fostering broader or earlier research dissemination across multiple projects, compared to systems potentially focused on fewer, more comprehensive projects culminating in full publications. Such differing patterns in scholarly output may have distinct implications for trainees' career progression and academic recognition in each region and warrant further investigation into their underlying causes and long-term effects.

While our survey did not specifically categorise training programmes as purely clinical vs. combined clinical and research tracks, it did capture the percentage of time trainees dedicated to research. U.S. trainees reported dedicating a significantly greater proportion of their training time to research activities compared to their European counterparts (median 30% vs. 17.5%, respectively). This finding suggests that a higher prevalence of research-intensive training pathways consuming more time is an unlikely explanation for the longer overall training durations observed in Europe. Instead, these longer durations are more directly attributable to the characteristic European training structure, as previously discussed. Despite comparable publication rates, both regions lacked robust formal mentorship frameworks, though European trainees faced more acute mentor shortages, likely exacerbated by limited faculty resources and institutional prioritisation compared to the U.S., where mentorship committees were more accessible.

Europe’s strong abstract productivity, despite fewer formal resources, suggests informal peer networks and role modelling may compensate for structural gaps,^24^ which aligns with its emphasis on collaborative, cohort-based training. These disparities underscore how regional training philosophies mediate mentorship’s impact: structured U.S. programmes amplify mentor-driven productivity, while in Europe, role modeling by motivated mentors seems crucial in overcoming structural limitations. Addressing Europe’s challenges requires institutional investments in mentorship infrastructure tailored to its unique model, ensuring equitable support for trainees navigating resource-intensive, clinically oriented pathways.^25^ In addition, European mentorship faces significant challenges stemming from country-specific obstacles. Unequal access to academic funding, illustrated by the stark disparity between European countries, contributes to reduced scientific contributions and expert allocation, while limited access to collaborative resources further complicates standardisation of mentorship efforts.^26^^,^^27^ Initiatives like the ESE’s Observership Programme, established to foster equitable mentor access and enhance cross-institutional collaboration, offer promising steps toward addressing these disparities. Ultimately, culturally informed strategies such as leveraging peer networks and aligning mentorship with translational priorities could bridge gaps, enhancing outcomes while preserving regional training strengths.

Our findings reaffirm that mentorship is broadly perceived as beneficial across both cohorts, enhancing training satisfaction, academic productivity, career progression, and well-being.^5^^,^^7^^,^^9^^,^^28^ However, the mechanisms driving these outcomes diverge markedly between U.S. and European contexts, reflecting distinct cultural and structural priorities. In the U.S., mentors’ strengths in accommodating communication styles, coordinating with colleagues (a characteristic rated significantly higher for U.S. mentors and particularly pertinent given that U.S. trainees more frequently reported having multiple mentors), and addressing gender/cultural differences—coupled with robust support for work-life balance and personalised career paths—align with institutional frameworks prioritising diversity, equity, and mentee-centred holistic development. These practices likely underpin the strong associations observed between inspirational mentorship, tailored career guidance, and reduced burnout. Such outcomes resonate with individualistic academic cultures that emphasise personal agency and tailored support, where alignment with mentee expectations fosters satisfaction.

Conversely, European mentorship emphasises fostering independence, strategic goal-setting, and acknowledging professional contributions—practices congruent with hierarchical academic systems valuing autonomy and collaborative achievement. Here, structured guidance (e.g., goal-setting strategies linked to reduced burnout) and inspirational mentorship dominated outcomes, suggesting mentees prioritise role models who elevate aspirations while providing pragmatic support to navigate rigid career pathways. The stark contrast in mentorship satisfaction drivers—holistic support in the U.S. vs. charismatic leadership in Europe—highlights how regional norms shape expectations. These divergences suggest that effective mentorship must adapt to local academic ecosystems: U.S. models integrate personal and professional development, while European success hinges on balancing inspirational leadership with methodical guidance.

Cultivating mentor competencies—such as active listening, trust-building, and work-life balance advocacy—should underpin training programmes,^29^, ^30^, ^31^ as these attributes were strongly linked to reduced burnout and career satisfaction in both cohorts. For Europe, integrating U.S.-inspired cultural competency initiatives could address gaps in accommodating gender and cultural differences, while U.S. programmes could formalise training to scale these strengths system-wide. Leveraging regional strengths is equally critical: U.S. institutions should expand mentor databases to preserve trainee autonomy in self-selecting relationships, while Europe could mitigate faculty shortages through cross-institutional networks, such as partnerships with local, national, regional, and international scientific societies, to pool mentorship resources and align with its collaborative training ethos. Hybrid frameworks combining Europe’s frequent interaction schedules with the U.S.’s holistic support—supported by virtual platforms—could bridge engagement gaps. Future longitudinal studies should evaluate these strategies while clarifying causal pathways, ensuring mentorship evolves to meet the dynamic demands of endocrinology.

There are several limitations to our study that warrant consideration. First, self-reported outcomes carry the risk of social desirability and recall bias. Second, our recruitment strategies, utilising professional society mailing lists and social media, may overrepresent trainees already engaged in active mentorship networks or research activities, including those dedicating a significant portion of their time to research. Similarly, we also acknowledge that our U.S. sample, with a median of 30% time dedicated to research, may reflect a higher engagement in research activities than the average across all U.S. endocrinology fellowship programmes. Both of these representativeness limitations should be considered when generalising findings to all training programme types, such as those that are primarily clinical. Third, structural differences between U.S. fellowship and European residency training models complicate direct comparisons, and the cross-sectional design precludes causal inference. Fourth, while this study provides comparative data on trainees with and without mentors, it did not conduct an in-depth qualitative exploration or employ highly specific survey items to detail the full spectrum of goals (e.g., specific clinical, research, or work-life balance objectives), satisfaction levels concerning these distinct goals, or a comprehensive assessment of the perceived need for mentorship among the subgroup of trainees who did not have a mentor. Obtaining such granular perspectives from unmentored individuals could offer valuable insights for future research aimed at understanding and addressing their specific support needs. Fifth, this study did not specifically investigate the details of research funding mechanisms available to trainees in both regions, nor their direct impact on the duration of individual training programmes. A comparative analysis of the availability and competitiveness of research funding sources for trainees, and the specific impact of such funding on individual training duration, was beyond the scope of our questionnaire and primary data collection but is worth future investigation.

Further limitations related to the specific of our European sample and the scope of certain inquiries. The inability to determine a precise denominator for the European trainee population due to diverse training structures limits our ability to quantify the exact representativeness of the European sample. Indeed, a further significant limitation is the treatment of Europe as a monolithic entity for analytical purposes. We acknowledge that training programmes, healthcare systems, and mentorship practices vary considerably across different European countries and specific subregions (e.g., Western, Eastern, Northern, Southern Europe). While our study included participants from a range of European nations, the sample size per country and for distinct subregions was insufficient to permit robust stratified analyses. Consequently, our aggregated ‘European’ findings may obscure important variations and should be interpreted with caution regarding their direct applicability to any single national or subregional context within Europe. Nevertheless, the study boasts several strengths that support its findings. Its novel, comprehensive approach provides the first in-depth comparison of mentorship practices across two distinct regions, employing a rigorously designed questionnaire based on validated mentoring competency tools that was adapted to account for regional nuances. Additionally, leveraging diverse recruitment channels allowed for a broader and more representative sample, while robust statistical analyses—including both univariable and multivariable methods—strengthen the associations observed between mentor characteristics and trainee outcomes. These strengths not only underscore the validity of our results but also provide a valuable framework for interpreting regional differences in mentorship, despite the inherent limitations.

This study underscores the universal value of mentorship in endocrinology training—enhancing academic productivity, career progression, and well-being—while revealing marked regional differences. U.S. trainees benefit from self-selected, personalised mentor relationships that offer holistic support and structured research opportunities, whereas European trainees engage in residency-embedded, frequent interactions that foster efficiency and independence despite challenges like limited formal infrastructure and mentor shortages. U.S. mentorship emphasises diversity-oriented, mentee-centred strategies that help reduce burnout, while European productivity stems from inspirational leadership and collaborative efficiency, reflecting more hierarchical, autonomy-driven systems. To address these disparities, adopting regionally tailored strategies that both build on local strengths and incorporate successful practices from other regions may help harmonise mentorship approaches, ultimately advancing academic excellence, career satisfaction, and clinical innovation in endocrinology.

## Contributors

DTT, HB, AFP, EJA, ASS, LVM, and IB conceptualised and designed the study. DTT, AS, LM, and IB collected the data. DTT, IB, AFP, and EJA analysed the data. DTT wrote the initial manuscript draft. DTT and IB accessed and verified the underlying data. All authors edited the manuscript, reviewed the manuscript for intellectual content, and approved the final version. All authors had full access to all the data in the study and had final responsibility for the decision to submit for publication.

## Data sharing statement

The de-identified individual participant data collected for this study, including data dictionaries, will be made available to qualified researchers upon reasonable request. Proposals should be directed to the corresponding author (Bancos.irina@mayo.edu). To gain access, data requestors will need to sign a data access agreement and provide a formal research proposal for review by the study investigators. The statistical analysis code used for this study is also available from the corresponding author upon reasonable request.

## Declaration of interests

IB reports serving as a deputy editor for the European Journal of Endocrinology. She has received consulting fees paid to her institution from Camurus, Crinetics, Xeris, Novonordisk, Astrazeneca, Diurnal, Neurocrine, Recordati, HRA pharma, Adrenas, Spruce, Sparrow, and Adaptyx. Her institution has also received grants or contracts from HRA pharma and Recordati. Additionally, Dr. Bancos has participated on an advisory board for Adrenas with payment to her institution and received personal travel support from HRA Pharma. DTT is a consultant for Immunovant. The other authors declare no competing interests. All authors confirm that these relationships are outside the scope of the submitted work on mentorship in endocrinology training.

## Appendix A Supplementary data

Supplement eTables S1–S5
