# Supplementary material for: Mentorship in endocrinology training: a cross-sectional study of the United States and Europe
Source: eClinicalMedicine. 2025 Jul 22;86:103377. doi: 10.1016/j.eclinm.2025.103377 (PMC12304690; doi:10.1016/j.eclinm.2025.103377)
Supplement: Supplement eTables S1–S5 [file mmc1.docx]

**Supplemental tables**

**eTable 1. Comparison of demographics, training satisfaction, and academic outcomes between Trainees with and without mentors in the Unites States and Europe**

|  | Europe | | | |  | US | | | |
| --- | --- | --- | --- | --- | --- | --- | --- | --- | --- |
|  | Has a mentor | |  | |  | Has a mentor | |  | |
|  | Yes (N=65) | No  (N=31) | Total (N=96) | P-value |  | Yes (N=110) | No  (N=44) | Total (N=154) | P-value |
| **Sex**, n (%) |  |  |  | 0.1425^1^ |  |  |  |  | 0.4716^1^ |
| Woman | 41 (63.1%) | 25 (80.6%) | 66 (68.8%) |  |  | 75 (68.2%) | 34 (77.3%) | 109 (70.8%) |  |
| Man | 23 (35.4%) | 5 (16.1%) | 28 (29.2%) |  |  | 34 (30.9%) | 10 (22.7%) | 44 (28.6%) |  |
| Prefer not to answer | 1 (1.5%) | 1 (3.2%) | 2 (2.1%) |  |  | 1 (0.9%) | 0 (0.0%) | 1 (0.6%) |  |
| **Race**, n (%) |  |  |  | 0.3332^1^ |  |  |  |  | 0.8221^1^ |
| Asian | 2 (3.1%) | 3 (9.7%) | 5 (5.2%) |  |  | 38 (34.5%) | 18 (40.9%) | 56 (36.4%) |  |
| Black or African American | 0 (0.0%) | 0 (0.0%) | 0 (0.0%) |  |  | 5 (4.5%) | 1 (2.3%) | 6 (3.9%) |  |
| White | 62 (95.4%) | 27 (87.1%) | 89 (92.7%) |  |  | 52 (47.3%) | 20 (45.5%) | 72 (46.8%) |  |
| Prefer not to answer | 1 (1.5%) | 1 (3.2%) | 2 (2.1%) |  |  | 15 (13.6%) | 5 (11.4%) | 20 (13.0%) |  |
| **Age** |  |  |  | 0.5152^2^ |  |  |  |  | 0.5457^2^ |
| Mean (SD) | 30.9 (4.13) | 32.4 (5.90) | 31.4 (4.80) |  |  | 33.8 (2.91) | 34.4 (3.74) | 33.9 (3.16) |  |
| Median | 30.0 | 31.0 | 30.0 |  |  | 33.0 | 34.0 | 33.0 |  |
| Q1, Q3 | 28.0, 32.0 | 28.0, 35.0 | 28.0, 32.0 |  |  | 32.0, 36.0 | 32.0, 35.0 | 32.0, 36.0 |  |
| **Number of years in training after completion of medical school**, n (%) |  |  |  | 0.5686^1^ |  |  |  |  | 1.0000^1^ |
| Less than 4 | 38 (58.5%) | 14 (45.2%) | 52 (54.2%) |  |  | 100 (90.9%) | 40 (90.9%) | 140 (90.9%) |  |
| 4 | 7 (10.8%) | 5 (16.1%) | 12 (12.5%) |  |  | 10 (9.1%) | 4 (9.1%) | 14 (9.1%) |  |
| 5 | 11 (16.9%) | 8 (25.8%) | 19 (19.8%) |  |  | 0 (0.0%) | 0 (0.0%) | 0 (0.0%) |  |
| 6 | 9 (13.8%) | 4 (12.9%) | 13 (13.5%) |  |  | 0 (0.0%) | 0 (0.0%) | 0 (0.0%) |  |
| **Endocrinology training structure***, n (%) |  |  |  | 0.5635^1^ |  |  |  |  |  |
| Internal medicine residency followed by subspecialization in Endocrinology | 23 (35.4%) | 14 (46.7%) | 37 (38.9%) |  |  | N/A | N/A | N/A |  |
| Residency in endocrinology and metabolic disease | 40 (61.5%) | 15 (50.0%) | 55 (57.9%) |  |  | N/A | N/A | N/A |  |
| Other | 2 (3.1%) | 1 (3.3%) | 3 (3.2%) |  |  | N/A | N/A | N/A |  |
| **Duration of endocrinology training program****, n (%) |  |  |  |  |  |  |  |  | 0.0783^1^ |
| 2 years | N/A | N/A | N/A |  |  | 68 (63.0%) | 35 (81.4%) | 103 (68.2%) |  |
| 3 years | N/A | N/A | N/A |  |  | 31 (28.7%) | 7 (16.3%) | 38 (25.2%) |  |
| More than 3 years | N/A | N/A | N/A |  |  | 9 (8.3%) | 1 (2.3%) | 10 (6.6%) |  |
| **Number of endocrine trainees in program** |  |  |  | 0.4268^2^ |  |  |  |  | 0.1606^2^ |
| Mean (SD) | 16.3 (12.09) | 13.9 (10.78) | 15.5 (11.68) |  |  | 6.6 (6.83) | 5.4 (2.56) | 6.3 (6.00) |  |
| Median | 11.5 | 10.0 | 11.0 |  |  | 6.0 | 5.0 | 6.0 |  |
| Q1, Q3 | 6.0, 28.0 | 4.0, 20.0 | 6.0, 28.0 |  |  | 4.0, 8.0 | 4.0, 7.0 | 4.0, 8.0 |  |
| **Research involvement**, n (%) |  |  |  | 0.1166^1^ |  |  |  |  | 0.0624^1^ |
| No | 11 (16.9%) | 11 (35.5%) | 22 (22.9%) |  |  | 19 (17.3%) | 16 (37.2%) | 35 (22.9%) |  |
| Yes - Clinical | 41 (63.1%) | 12 (38.7%) | 53 (55.2%) |  |  | 72 (65.5%) | 22 (51.2%) | 94 (61.4%) |  |
| Yes - Basic | 6 (9.2%) | 3 (9.7%) | 9 (9.4%) |  |  | 14 (12.7%) | 3 (7.0%) | 17 (11.1%) |  |
| Yes - Translational | 7 (10.8%) | 5 (16.1%) | 12 (12.5%) |  |  | 5 (4.5%) | 2 (4.7%) | 7 (4.6%) |  |
| **Percentage of training dedicated to research** |  |  |  | 0.0346^2^ |  |  |  |  | 0.0005^2^ |
| Mean (SD) | 22.8 (18.92) | 16.8 (22.66) | 20.9 (20.22) |  |  | 35.6 (22.83) | 21.7 (19.88) | 31.7 (22.85) |  |
| Median | 20.0 | 10.0 | 17.5 |  |  | 30.0 | 15.0 | 30.0 |  |
| Q1, Q3 | 10.0, 30.0 | 0.0, 25.0 | 5.0, 30.0 |  |  | 20.0, 50.0 | 10.0, 33.0 | 10.0, 50.0 |  |
| **Number of publications** |  |  |  | 0.5302^2^ |  |  |  |  | 0.0239^2^ |
| Mean (SD) | 9.1 (31.12) | 13.4 (47.24) | 10.5 (36.88) |  |  | 3.8 (5.07) | 2.0 (1.99) | 3.3 (4.52) |  |
| Median | 2.0 | 2.0 | 2.0 |  |  | 2.0 | 2.0 | 2.0 |  |
| Q1, Q3 | 1.0, 7.0 | 0.0, 6.0 | 0.0, 7.0 |  |  | 1.0, 4.0 | 0.0, 3.0 | 1.0, 4.0 |  |
| **Number of abstracts, posters, and oral presentations** |  |  |  | 0.8037^2^ |  |  |  |  | 0.0124^2^ |
| Mean (SD) | 10.4 (15.37) | 18.6 (54.61) | 12.9 (33.06) |  |  | 6.2 (6.43) | 4.0 (3.79) | 5.6 (5.89) |  |
| Median | 5.0 | 6.0 | 6.0 |  |  | 5.0 | 3.0 | 4.0 |  |
| Q1, Q3 | 3.0, 12.0 | 2.0, 15.0 | 3.0, 14.0 |  |  | 2.0, 8.0 | 2.0, 5.0 | 2.0, 7.0 |  |
| **Previously applied for a grant**, n (%) |  |  |  | 0.6647^1^ |  |  |  |  | 0.0104^1^ |
| Yes | 23 (35.4%) | 12 (40.0%) | 35 (36.8%) |  |  | 32 (29.4%) | 4 (9.5%) | 36 (23.8%) |  |
| No | 42 (64.6%) | 18 (60.0%) | 60 (63.2%) |  |  | 77 (70.6%) | 38 (90.5%) | 115 (76.2%) |  |
| **Academic appointment**, n (%) |  |  |  | 0.7975^1^ |  |  |  |  | 0.2342^1^ |
| None | 52 (80.0%) | 22 (71.0%) | 74 (77.1%) |  |  | 78 (71.6%) | 34 (79.1%) | 112 (73.7%) |  |
| Clinical Instructor | 2 (3.1%) | 1 (3.2%) | 3 (3.1%) |  |  | 8 (7.3%) | 4 (9.3%) | 12 (7.9%) |  |
| Assistant Professor | 4 (6.2%) | 2 (6.5%) | 6 (6.3%) |  |  | 21 (19.3%) | 3 (7.0%) | 24 (15.8%) |  |
| Associate Professor | 2 (3.1%) | 1 (3.2%) | 3 (3.1%) |  |  | 0 (0.0%) | 0 (0.0%) | 0 (0.0%) |  |
| Other | 5 (7.7%) | 5 (16.1%) | 10 (10.4%) |  |  | 2 (1.8%) | 2 (4.7%) | 4 (2.6%) |  |
| **Satisfaction with endocrinology training**, n (%) |  |  |  | 0.0007^1^ |  |  |  |  | 0.0072^1^ |
| Very Satisfied | 18 (27.7%) | 3 (9.7%) | 21 (21.9%) |  |  | 51 (46.4%) | 11 (25.0%) | 62 (40.3%) |  |
| Satisfied | 30 (46.2%) | 6 (19.4%) | 36 (37.5%) |  |  | 50 (45.5%) | 20 (45.5%) | 70 (45.5%) |  |
| Neutral | 11 (16.9%) | 13 (41.9%) | 24 (25.0%) |  |  | 6 (5.5%) | 7 (15.9%) | 13 (8.4%) |  |
| Dissatisfied | 6 (9.2%) | 7 (22.6%) | 13 (13.5%) |  |  | 2 (1.8%) | 4 (9.1%) | 6 (3.9%) |  |
| Very Dissatisfied | 0 (0.0%) | 2 (6.5%) | 2 (2.1%) |  |  | 1 (0.9%) | 2 (4.5%) | 3 (1.9%) |  |
| **Future type of practice intent**, n (%) |  |  |  |  |  |  |  |  |  |
| Academic | 41 (63.1%) | 20 (64.5%) | 61 (63.5%) | 0.8910^1^ |  | 76 (69.1%) | 22 (50.0%) | 98 (63.6%) | 0.0261^1^ |
| Private | 22 (33.8%) | 13 (41.9%) | 35 (36.5%) | 0.4413^1^ |  | 43 (39.1%) | 24 (54.5%) | 67 (43.5%) | 0.0805^1^ |
| Industry | 2 (3.1%) | 2 (6.5%) | 4 (4.2%) | 0.4391^1^ |  | 7 (6.4%) | 6 (13.6%) | 13 (8.4%) | 0.1425^1^ |
| Research | 37 (56.9%) | 15 (48.4%) | 52 (54.2%) | 0.4325^1^ |  | 14 (12.7%) | 7 (15.9%) | 21 (13.6%) | 0.6032^1^ |
| Clinical | 59 (90.8%) | 23 (74.2%) | 82 (85.4%) | 0.0314^1^ |  | 54 (49.1%) | 24 (54.5%) | 78 (50.6%) | 0.5408^1^ |
| Education scholar | 11 (16.9%) | 4 (12.9%) | 15 (15.6%) | 0.6120^1^ |  | 16 (14.5%) | 6 (13.6%) | 22 (14.3%) | 0.8842^1^ |
| Other | 1 (1.5%) | 0 (0.0%) | 1 (1.0%) | 0.4875^1^ |  | 2 (1.8%) | 1 (2.3%) | 3 (1.9%) | 0.8537^1^ |
| Unsure | 1 (1.5%) | 1 (3.2%) | 2 (2.1%) | 0.5883^1^ |  | 6 (5.5%) | 1 (2.3%) | 7 (4.5%) | 0.3918^1^ |
| **Formal mentorship programs available at their institution**, n (%) |  |  |  | 0.0110^1^ |  |  |  |  | 0.0048^1^ |
| Yes | 25 (39.7%) | 1 (6.3%) | 26 (32.9%) |  |  | 52 (47.3%) | 9 (22.0%) | 61 (40.4%) |  |
| No | 38 (60.3%) | 15 (93.8%) | 53 (67.1%) |  |  | 58 (52.7%) | 32 (78.0%) | 90 (59.6%) |  |
| **Mentorship program available**, n (%) |  |  |  |  |  |  |  |  |  |
| Mentorship committee | 1 (1.5%) | 0 (0.0%) | 1 (1.0%) | 0.4875^1^ |  | 16 (14.5%) | 1 (2.3%) | 17 (11.0%) | 0.0281^1^ |
| IDP | 8 (12.3%) | 1 (3.2%) | 9 (9.4%) | 0.1534^1^ |  | 19 (17.3%) | 5 (11.4%) | 24 (15.6%) | 0.3611^1^ |
| Standardized program | 15 (23.1%) | 0 (0.0%) | 15 (15.6%) | 0.0036^1^ |  | 25 (22.7%) | 2 (4.5%) | 27 (17.5%) | 0.0073^1^ |
| Other | 4 (6.2%) | 0 (0.0%) | 4 (4.2%) | 0.1583^1^ |  | 7 (6.4%) | 1 (2.3%) | 8 (5.2%) | 0.3014^1^ |
| **Barriers to finding a mentor**, n (%) |  |  |  | <.0001^1^ |  |  |  |  | 0.0008^1^ |
| Yes | 8 (12.3%) | 11 (68.8%) | 19 (23.5%) |  |  | 12 (11.0%) | 14 (34.1%) | 26 (17.3%) |  |
| No | 57 (87.7%) | 5 (31.3%) | 62 (76.5%) |  |  | 97 (89.0%) | 27 (65.9%) | 124 (82.7%) |  |
| **Type of barriers encountered in finding a mentor**, n (%) |  |  |  | 0.3251^1^ |  |  |  |  | 0.5406^1^ |
| Limited mentorship opportunities | 1 (33.3%) | 4 (44.4%) | 5 (41.7%) |  |  | 4 (40.0%) | 6 (46.2%) | 10 (43.5%) |  |
| Insufficient mentor engagement | 1 (33.3%) | 4 (44.4%) | 5 (41.7%) |  |  | 5 (50.0%) | 4 (30.8%) | 9 (39.1%) |  |
| Personal barriers or mentee hesitation | 0 (0.0%) | 1 (11.1%) | 1 (8.3%) |  |  | 1 (10.0%) | 1 (7.7%) | 2 (8.7%) |  |
| Communication or cultural barriers | 1 (33.3%) | 0 (0.0%) | 1 (8.3%) |  |  | 0 (0.0%) | 2 (15.4%) | 2 (8.7%) |  |
| **Sufficient availability of faculty that could serve as mentors at their institution**, n (%) |  |  |  | 0.0099^1^ |  |  |  |  | 0.0812^1^ |
| Yes | 46 (71.9%) | 6 (37.5%) | 52 (65.0%) |  |  | 98 (89.1%) | 32 (78.0%) | 130 (86.1%) |  |
| No | 18 (28.1%) | 10 (62.5%) | 28 (35.0%) |  |  | 12 (10.9%) | 9 (22.0%) | 21 (13.9%) |  |
| ^1^Chi-Square p-value; ^2^Kruskal-Wallis p-value;  *For U.S. respondents, this question was not applicable as all trainees follow a standardized pathway of internal medicine residency followed by subspecialization in endocrinology, as described in the text  ** For European respondents, this question was not applicable due to the greater heterogeneity and different overall structure of their training programs  N/A: Not applicable | | | | | | | | | |

**eTable 2. Mentor characteristics among those who reported having a mentor**

|  | Europe (N=65) | US (N=110) | Total (N=175) | P-value |
| --- | --- | --- | --- | --- |
| **Active listener**, n (%) |  |  |  | 0.2591^1^ |
| Agree | 46 (79.3%) | 94 (87.0%) | 140 (84.3%) |  |
| Neutral | 9 (15.5%) | 8 (7.4%) | 17 (10.2%) |  |
| Disagree | 3 (5.2%) | 6 (5.6%) | 9 (5.4%) |  |
| **Provides helpful feedback and coaching on my performance**, n (%) |  |  |  | 0.3309^1^ |
| Agree | 47 (81.0%) | 92 (86.0%) | 139 (84.2%) |  |
| Neutral | 9 (15.5%) | 9 (8.4%) | 18 (10.9%) |  |
| Disagree | 2 (3.4%) | 6 (5.6%) | 8 (4.8%) |  |
| **Develops a trusting relationship**, n (%) |  |  |  | 0.9116^1^ |
| Agree | 48 (82.8%) | 92 (85.2%) | 140 (84.3%) |  |
| Neutral | 6 (10.3%) | 10 (9.3%) | 16 (9.6%) |  |
| Disagree | 4 (6.9%) | 6 (5.6%) | 10 (6.0%) |  |
| **Is accommodating to different communication styles**, n (%) |  |  |  | 0.0422^1^ |
| Agree | 39 (67.2%) | 88 (81.5%) | 127 (76.5%) |  |
| Neutral | 16 (27.6%) | 13 (12.0%) | 29 (17.5%) |  |
| Disagree | 3 (5.2%) | 7 (6.5%) | 10 (6.0%) |  |
| **Pursues strategies to improve communication**, n (%) |  |  |  | 0.3583^1^ |
| Agree | 38 (65.5%) | 82 (75.9%) | 120 (72.3%) |  |
| Neutral | 12 (20.7%) | 16 (14.8%) | 28 (16.9%) |  |
| Disagree | 8 (13.8%) | 10 (9.3%) | 18 (10.8%) |  |
| **Coordinates and collaborates with other mentors**, n (%) |  |  |  | 0.0018^1^ |
| Agree | 32 (55.2%) | 86 (80.4%) | 118 (71.5%) |  |
| Neutral | 14 (24.1%) | 14 (13.1%) | 28 (17.0%) |  |
| Disagree | 12 (20.7%) | 7 (6.5%) | 19 (11.5%) |  |
| **Sets clear mentorship expectations**, n (%) |  |  |  | 0.1309^1^ |
| Agree | 32 (55.2%) | 74 (69.8%) | 106 (64.6%) |  |
| Neutral | 13 (22.4%) | 19 (17.9%) | 32 (19.5%) |  |
| Disagree | 13 (22.4%) | 13 (12.3%) | 26 (15.9%) |  |
| **Aligns with your mentorship expectations**, n (%) |  |  |  | 0.0729^1^ |
| Agree | 39 (67.2%) | 88 (82.2%) | 127 (77.0%) |  |
| Neutral | 8 (13.8%) | 10 (9.3%) | 18 (10.9%) |  |
| Disagree | 11 (19.0%) | 9 (8.4%) | 20 (12.1%) |  |
| **Is considerate about the mentor-mentee differences**, n (%) |  |  |  | 0.0018^1^ |
| Agree | 37 (63.8%) | 91 (84.3%) | 128 (77.1%) |  |
| Neutral | 16 (27.6%) | 8 (7.4%) | 24 (14.5%) |  |
| Disagree | 5 (8.6%) | 9 (8.3%) | 14 (8.4%) |  |
| **Sets clear goals**, n (%) |  |  |  | 0.0580^1^ |
| Agree | 34 (58.6%) | 82 (75.9%) | 116 (69.9%) |  |
| Neutral | 12 (20.7%) | 15 (13.9%) | 27 (16.3%) |  |
| Disagree | 12 (20.7%) | 11 (10.2%) | 23 (13.9%) |  |
| **Helps me develop strategies to meet my goals**, n (%) |  |  |  | 0.0027^1^ |
| Agree | 33 (57.9%) | 89 (82.4%) | 122 (73.9%) |  |
| Neutral | 13 (22.8%) | 9 (8.3%) | 22 (13.3%) |  |
| Disagree | 11 (19.3%) | 10 (9.3%) | 21 (12.7%) |  |
| **Estimates my knowledge, abilities and skills**, n (%) |  |  |  | 0.0376^1^ |
| Agree | 40 (69.0%) | 92 (85.2%) | 132 (79.5%) |  |
| Neutral | 11 (19.0%) | 8 (7.4%) | 19 (11.4%) |  |
| Disagree | 7 (12.1%) | 8 (7.4%) | 15 (9.0%) |  |
| **Enhances my knowledge, abilities and skills**, n (%) |  |  |  | 0.2067^1^ |
| Agree | 44 (77.2%) | 92 (87.6%) | 136 (84.0%) |  |
| Neutral | 8 (14.0%) | 7 (6.7%) | 15 (9.3%) |  |
| Disagree | 5 (8.8%) | 6 (5.7%) | 11 (6.8%) |  |
| **Inspires me and motivates me to meet my goals, and maximize my potential**, n (%) |  |  |  | 0.3581^1^ |
| Agree | 44 (75.9%) | 90 (84.9%) | 134 (81.7%) |  |
| Neutral | 8 (13.8%) | 9 (8.5%) | 17 (10.4%) |  |
| Disagree | 6 (10.3%) | 7 (6.6%) | 13 (7.9%) |  |
| **Empowers me to do my job**, n (%) |  |  |  | 0.2630^1^ |
| Agree | 42 (72.4%) | 89 (83.2%) | 131 (79.4%) |  |
| Neutral | 10 (17.2%) | 11 (10.3%) | 21 (12.7%) |  |
| Disagree | 6 (10.3%) | 7 (6.5%) | 13 (7.9%) |  |
| **Stimulates my creative thinking**, n (%) |  |  |  | 0.1286^1^ |
| Agree | 41 (73.2%) | 90 (84.9%) | 131 (80.9%) |  |
| Neutral | 7 (12.5%) | 10 (9.4%) | 17 (10.5%) |  |
| Disagree | 8 (14.3%) | 6 (5.7%) | 14 (8.6%) |  |
| **Acknowledges my professional contributions and gives me appropriate credit**, n (%) |  |  |  | 0.4848^1^ |
| Agree | 45 (77.6%) | 91 (85.0%) | 136 (82.4%) |  |
| Neutral | 8 (13.8%) | 10 (9.3%) | 18 (10.9%) |  |
| Disagree | 5 (8.6%) | 6 (5.6%) | 11 (6.7%) |  |
| **Fosters my independence**, n (%) |  |  |  | 0.2600^1^ |
| Agree | 44 (75.9%) | 92 (86.0%) | 136 (82.4%) |  |
| Neutral | 7 (12.1%) | 8 (7.5%) | 15 (9.1%) |  |
| Disagree | 7 (12.1%) | 7 (6.5%) | 14 (8.5%) |  |
| **Accounts for differences in my sex and cultural background**, n (%) |  |  |  | <.0001^1^ |
| Agree | 23 (39.7%) | 80 (74.8%) | 103 (62.4%) |  |
| Neutral | 25 (43.1%) | 22 (20.6%) | 47 (28.5%) |  |
| Disagree | 10 (17.2%) | 5 (4.7%) | 15 (9.1%) |  |
| **Helps me network effectively**, n (%) |  |  |  | 0.0237^1^ |
| Agree | 34 (58.6%) | 84 (78.5%) | 118 (71.5%) |  |
| Neutral | 17 (29.3%) | 15 (14.0%) | 32 (19.4%) |  |
| Disagree | 7 (12.1%) | 8 (7.5%) | 15 (9.1%) |  |
| **Helps me develop a personalized career path that aligns with my goals**, n (%) |  |  |  | 0.0399^1^ |
| Agree | 35 (61.4%) | 86 (79.6%) | 121 (73.3%) |  |
| Neutral | 13 (22.8%) | 14 (13.0%) | 27 (16.4%) |  |
| Disagree | 9 (15.8%) | 8 (7.4%) | 17 (10.3%) |  |
| **Helps me achieve an adequate work/life balance**, n (%) |  |  |  | 0.0003^1^ |
| Agree | 25 (43.1%) | 80 (74.1%) | 105 (63.3%) |  |
| Neutral | 20 (34.5%) | 20 (18.5%) | 40 (24.1%) |  |
| Disagree | 13 (22.4%) | 8 (7.4%) | 21 (12.7%) |  |
| **Is my role model**, n (%) |  |  |  | 0.3083^1^ |
| Agree | 35 (61.4%) | 77 (72.0%) | 112 (68.3%) |  |
| Neutral | 12 (21.1%) | 19 (17.8%) | 31 (18.9%) |  |
| Disagree | 10 (17.5%) | 11 (10.3%) | 21 (12.8%) |  |
| **Has professional and mentoring experience that facilitates my development**, n (%) |  |  |  | 0.0951^1^ |
| Agree | 41 (73.2%) | 91 (84.3%) | 132 (80.5%) |  |
| Neutral | 12 (21.4%) | 10 (9.3%) | 22 (13.4%) |  |
| Disagree | 3 (5.4%) | 7 (6.5%) | 10 (6.1%) |  |
| **Facilitates access to research and professional resources**, n (%) |  |  |  | 0.2317^1^ |
| Agree | 40 (69.0%) | 87 (80.6%) | 127 (76.5%) |  |
| Neutral | 12 (20.7%) | 13 (12.0%) | 25 (15.1%) |  |
| Disagree | 6 (10.3%) | 8 (7.4%) | 14 (8.4%) |  |
| ^1^Chi-Square p-value; | | | | |

**eTable 3. Association between mentor characteristics and academic productivity, mentorship satisfaction, and burnout among those reported to have had a mentor in the United States Cohort**

|  | | Experienced increased academic productivity | | | | | Satisfied by mentorship relationship | | | | | Experienced less burnout and stress | | | |
| --- | --- | --- | --- | --- | --- | --- | --- | --- | --- | --- | --- | --- | --- | --- | --- |
|  | | Yes (N=91) | | No (N=18) | P-value | | Yes (N=94) | | No (N=15) | P-value | | Yes (N=66) | No (N=43) | | P-value |
| **Active listener**, n (%) | |  | |  | <.0001^1^ | |  | |  | <.0001^1^ | |  |  | | 0.1557^1^ |
| Agree | | 84 (93.3%) | | 10 (55.6%) |  | | 86 (92.5%) | | 8 (53.3%) |  | | 59 (90.8%) | 35 (81.4%) | |  |
| Do Not Agree | | 6 (6.7%) | | 8 (44.4%) |  | | 7 (7.5%) | | 7 (46.7%) |  | | 6 (9.2%) | 8 (18.6%) | |  |
| **Provides helpful feedback and coaching on my performance**, n (%) | |  | |  | <.0001^1^ | |  | |  | <.0001^1^ | |  |  | | 0.0047^1^ |
| Agree | | 83 (93.3%) | | 9 (50.0%) |  | | 86 (93.5%) | | 6 (40.0%) |  | | 60 (93.8%) | 32 (74.4%) | |  |
| Do Not Agree | | 6 (6.7%) | | 9 (50.0%) |  | | 6 (6.5%) | | 9 (60.0%) |  | | 4 (6.3%) | 11 (25.6%) | |  |
| **Develops a trusting relationship**, n (%) | |  | |  | <.0001^1^ | |  | |  | <.0001^1^ | |  |  | | 0.0018^1^ |
| Agree | | 83 (92.2%) | | 9 (50.0%) |  | | 87 (93.5%) | | 5 (33.3%) |  | | 61 (93.8%) | 31 (72.1%) | |  |
| Do Not Agree | | 7 (7.8%) | | 9 (50.0%) |  | | 6 (6.5%) | | 10 (66.7%) |  | | 4 (6.2%) | 12 (27.9%) | |  |
| **Is accommodating to different communication styles**, n (%) | |  | |  | <.0001^1^ | |  | |  | <.0001^1^ | |  |  | | 0.1243^1^ |
| Agree | | 80 (88.9%) | | 8 (44.4%) |  | | 82 (88.2%) | | 6 (40.0%) |  | | 56 (86.2%) | 32 (74.4%) | |  |
| Do Not Agree | | 10 (11.1%) | | 10 (55.6%) |  | | 11 (11.8%) | | 9 (60.0%) |  | | 9 (13.8%) | 11 (25.6%) | |  |
| **Pursues strategies to improve communication**, n (%) | |  | |  | <.0001^1^ | |  | |  | <.0001^1^ | |  |  | | 0.0326^1^ |
| Agree | | 76 (84.4%) | | 6 (33.3%) |  | | 78 (83.9%) | | 4 (26.7%) |  | | 54 (83.1%) | 28 (65.1%) | |  |
| Do Not Agree | | 14 (15.6%) | | 12 (66.7%) |  | | 15 (16.1%) | | 11 (73.3%) |  | | 11 (16.9%) | 15 (34.9%) | |  |
| **Coordinates and collaborates with other mentors**, n (%) | |  | |  | 0.0004^1^ | |  | |  | 0.0004^1^ | |  |  | | 0.0177^1^ |
| Agree | | 77 (86.5%) | | 9 (50.0%) |  | | 79 (85.9%) | | 7 (46.7%) |  | | 57 (87.7%) | 29 (69.0%) | |  |
| Do Not Agree | | 12 (13.5%) | | 9 (50.0%) |  | | 13 (14.1%) | | 8 (53.3%) |  | | 8 (12.3%) | 13 (31.0%) | |  |
| **Sets clear mentorship expectations**, n (%) | |  | |  | <.0001^1^ | |  | |  | <.0001^1^ | |  |  | | 0.0095^1^ |
| Agree | | 69 (78.4%) | | 5 (27.8%) |  | | 70 (76.9%) | | 4 (26.7%) |  | | 50 (79.4%) | 24 (55.8%) | |  |
| Do Not Agree | | 19 (21.6%) | | 13 (72.2%) |  | | 21 (23.1%) | | 11 (73.3%) |  | | 13 (20.6%) | 19 (44.2%) | |  |
| **Aligns with your mentorship expectations**, n (%) | |  | |  | <.0001^1^ | |  | |  | <.0001^1^ | |  |  | | 0.0041^1^ |
| Agree | | 81 (90.0%) | | 7 (41.2%) |  | | 85 (91.4%) | | 3 (21.4%) |  | | 59 (90.8%) | 29 (69.0%) | |  |
| Do Not Agree | | 9 (10.0%) | | 10 (58.8%) |  | | 8 (8.6%) | | 11 (78.6%) |  | | 6 (9.2%) | 13 (31.0%) | |  |
| **Is considerate about the mentor-mentee differences**, n (%) | |  | |  | 0.0002^1^ | |  | |  | <.0001^1^ | |  |  | | 0.0811^1^ |
| Agree | | 81 (90.0%) | | 10 (55.6%) |  | | 85 (91.4%) | | 6 (40.0%) |  | | 58 (89.2%) | 33 (76.7%) | |  |
| Do Not Agree | | 9 (10.0%) | | 8 (44.4%) |  | | 8 (8.6%) | | 9 (60.0%) |  | | 7 (10.8%) | 10 (23.3%) | |  |
| **Sets clear goals**, n (%) | |  | |  | <.0001^1^ | |  | |  | <.0001^1^ | |  |  | | 0.0326^1^ |
| Agree | | 75 (83.3%) | | 7 (38.9%) |  | | 77 (82.8%) | | 5 (33.3%) |  | | 54 (83.1%) | 28 (65.1%) | |  |
| Do Not Agree | | 15 (16.7%) | | 11 (61.1%) |  | | 16 (17.2%) | | 10 (66.7%) |  | | 11 (16.9%) | 15 (34.9%) | |  |
| **Helps me develop strategies to meet my goals**, n (%) | |  | |  | <.0001^1^ | |  | |  | <.0001^1^ | |  |  | | 0.0009^1^ |
| Agree | | 81 (90.0%) | | 8 (44.4%) |  | | 84 (90.3%) | | 5 (33.3%) |  | | 60 (92.3%) | 29 (67.4%) | |  |
| Do Not Agree | | 9 (10.0%) | | 10 (55.6%) |  | | 9 (9.7%) | | 10 (66.7%) |  | | 5 (7.7%) | 14 (32.6%) | |  |
| **Estimates my knowledge, abilities and skills**, n (%) | |  | |  | <.0001^1^ | |  | |  | <.0001^1^ | |  |  | | 0.0018^1^ |
| Agree | | 83 (92.2%) | | 9 (50.0%) |  | | 86 (92.5%) | | 6 (40.0%) |  | | 61 (93.8%) | 31 (72.1%) | |  |
| Do Not Agree | | 7 (7.8%) | | 9 (50.0%) |  | | 7 (7.5%) | | 9 (60.0%) |  | | 4 (6.2%) | 12 (27.9%) | |  |
| **Enhances my knowledge, abilities and skills**, n (%) | |  | |  | <.0001^1^ | |  | |  | <.0001^1^ | |  |  | | 0.0172^1^ |
| Agree | | 85 (95.5%) | | 7 (43.8%) |  | | 87 (94.6%) | | 5 (38.5%) |  | | 60 (93.8%) | 32 (78.0%) | |  |
| Do Not Agree | | 4 (4.5%) | | 9 (56.3%) |  | | 5 (5.4%) | | 8 (61.5%) |  | | 4 (6.3%) | 9 (22.0%) | |  |
| **Inspires me and motivates me to meet my goals, and maximize my potential**, n (%) | |  | |  | <.0001^1^ | |  | |  | <.0001^1^ | |  |  | | 0.0017^1^ |
| Agree | | 84 (94.4%) | | 6 (35.3%) |  | | 85 (92.4%) | | 5 (35.7%) |  | | 60 (93.8%) | 30 (71.4%) | |  |
| Do Not Agree | | 5 (5.6%) | | 11 (64.7%) |  | | 7 (7.6%) | | 9 (64.3%) |  | | 4 (6.3%) | 12 (28.6%) | |  |
| **Empowers me to do my job**, n (%) | |  | |  | <.0001^1^ | |  | |  | <.0001^1^ | |  |  | | 0.0004^1^ |
| Agree | | 82 (92.1%) | | 7 (38.9%) |  | | 84 (91.3%) | | 5 (33.3%) |  | | 60 (93.8%) | 29 (67.4%) | |  |
| Do Not Agree | | 7 (7.9%) | | 11 (61.1%) |  | | 8 (8.7%) | | 10 (66.7%) |  | | 4 (6.3%) | 14 (32.6%) | |  |
| **Stimulates my creative thinking**, n (%) | |  | |  | 0.0001^1^ | |  | |  | 0.0002^1^ | |  |  | | 0.0097^1^ |
| Agree | | 80 (90.9%) | | 10 (55.6%) |  | | 82 (90.1%) | | 8 (53.3%) |  | | 59 (92.2%) | 31 (73.8%) | |  |
| Do Not Agree | | 8 (9.1%) | | 8 (44.4%) |  | | 9 (9.9%) | | 7 (46.7%) |  | | 5 (7.8%) | 11 (26.2%) | |  |
| **Acknowledges my professional contributions and gives me appropriate credit**, n (%) | |  | |  | <.0001^1^ | |  | |  | <.0001^1^ | |  |  | | 0.0021^1^ |
| Agree | | 82 (92.1%) | | 9 (50.0%) |  | | 84 (91.3%) | | 7 (46.7%) |  | | 60 (93.8%) | 31 (72.1%) | |  |
| Do Not Agree | | 7 (7.9%) | | 9 (50.0%) |  | | 8 (8.7%) | | 8 (53.3%) |  | | 4 (6.3%) | 12 (27.9%) | |  |
| **Fosters my independence**, n (%) | |  | |  | <.0001^1^ | |  | |  | <.0001^1^ | |  |  | | 0.0241^1^ |
| Agree | | 82 (92.1%) | | 10 (55.6%) |  | | 84 (91.3%) | | 8 (53.3%) |  | | 59 (92.2%) | 33 (76.7%) | |  |
| Do Not Agree | | 7 (7.9%) | | 8 (44.4%) |  | | 8 (8.7%) | | 7 (46.7%) |  | | 5 (7.8%) | 10 (23.3%) | |  |
| **Accounts for differences in my gender and cultural background**, n (%) | |  | |  | 0.0080^1^ | |  | |  | 0.0069^1^ | |  |  | | 0.0052^1^ |
| Agree | | 71 (79.8%) | | 9 (50.0%) |  | | 73 (79.3%) | | 7 (46.7%) |  | | 54 (84.4%) | 26 (60.5%) | |  |
| Do Not Agree | | 18 (20.2%) | | 9 (50.0%) |  | | 19 (20.7%) | | 8 (53.3%) |  | | 10 (15.6%) | 17 (39.5%) | |  |
| **Helps me network effectively**, n (%) | |  | |  | <.0001^1^ | |  | |  | <.0001^1^ | |  |  | | 0.0012^1^ |
| Agree | | 79 (88.8%) | | 5 (27.8%) |  | | 80 (87.0%) | | 4 (26.7%) |  | | 57 (89.1%) | 27 (62.8%) | |  |
| Do Not Agree | | 10 (11.2%) | | 13 (72.2%) |  | | 12 (13.0%) | | 11 (73.3%) |  | | 7 (10.9%) | 16 (37.2%) | |  |
| **Helps me develop a personalized career path that aligns with my goals**, n (%) | |  | |  | <.0001^1^ | |  | |  | <.0001^1^ | |  |  | | 0.0004^1^ |
| Agree | | 79 (87.8%) | | 7 (38.9%) |  | | 83 (89.2%) | | 3 (20.0%) |  | | 59 (90.8%) | 27 (62.8%) | |  |
| Do Not Agree | | 11 (12.2%) | | 11 (61.1%) |  | | 10 (10.8%) | | 12 (80.0%) |  | | 6 (9.2%) | 16 (37.2%) | |  |
| **Helps me achieve an adequate work/life balance**, n (%) | |  | |  | <.0001^1^ | |  | |  | <.0001^1^ | |  |  | | <.0001^1^ |
| Agree | | 74 (82.2%) | | 6 (33.3%) |  | | 76 (81.7%) | | 4 (26.7%) |  | | 58 (89.2%) | 22 (51.2%) | |  |
| Do Not Agree | | 16 (17.8%) | | 12 (66.7%) |  | | 17 (18.3%) | | 11 (73.3%) |  | | 7 (10.8%) | 21 (48.8%) | |  |
| **Is my role model**, n (%) | |  | |  | <.0001^1^ | |  | |  | <.0001^1^ | |  |  | | <.0001^1^ |
| Agree | | 72 (80.9%) | | 5 (27.8%) |  | | 75 (81.5%) | | 2 (13.3%) |  | | 56 (87.5%) | 21 (48.8%) | |  |
| Do Not Agree | | 17 (19.1%) | | 13 (72.2%) |  | | 17 (18.5%) | | 13 (86.7%) |  | | 8 (12.5%) | 22 (51.2%) | |  |
| **Has professional and mentoring experience that facilitates my development**, n (%) | |  | |  | <.0001^1^ | |  | |  | <.0001^1^ | |  |  | | 0.0047^1^ |
| Agree | | 82 (91.1%) | | 9 (50.0%) |  | | 84 (90.3%) | | 7 (46.7%) |  | | 60 (92.3%) | 31 (72.1%) | |  |
| Do Not Agree | | 8 (8.9%) | | 9 (50.0%) |  | | 9 (9.7%) | | 8 (53.3%) |  | | 5 (7.7%) | 12 (27.9%) | |  |
| **Facilitates access to research and professional resources**, n (%) | |  | |  | <.0001^1^ | |  | |  | 0.0004^1^ | |  |  | | 0.0212^1^ |
| Agree | | 79 (87.8%) | | 8 (44.4%) |  | | 80 (86.0%) | | 7 (46.7%) |  | | 57 (87.7%) | 30 (69.8%) | |  |
| Do Not Agree | | 11 (12.2%) | | 10 (55.6%) |  | | 13 (14.0%) | | 8 (53.3%) |  | | 8 (12.3%) | 13 (30.2%) | |  |
| ^1^Chi-Square p-value; |  | |  | | |  | |  | | |  | | |  | |

**eTable 4. Association between mentor characteristics and academic productivity, mentorship satisfaction, and burnout among those reported to have had a mentor in the European Cohort**

|  | | Experienced increased academic productivity | | | | | Satisfaction with mentorship relationship | | | | | Experienced less burnout and stress | | | |
| --- | --- | --- | --- | --- | --- | --- | --- | --- | --- | --- | --- | --- | --- | --- | --- |
|  | | Yes (N=52) | | No (N=13) | P-value | | Yes (N=49) | | No (N=15) | P-value | | Yes (N=28) | No (N=37) | | P-value |
| **Active listener**, n (%) | |  | |  | 0.3084^1^ | |  | |  | 0.0001^1^ | |  |  | | 0.0928^1^ |
| Agree | | 37 (82.2%) | | 9 (69.2%) |  | | 39 (90.7%) | | 6 (42.9%) |  | | 24 (88.9%) | 22 (71.0%) | |  |
| Do Not Agree | | 8 (17.8%) | | 4 (30.8%) |  | | 4 (9.3%) | | 8 (57.1%) |  | | 3 (11.1%) | 9 (29.0%) | |  |
| **Provides helpful feedback and coaching on my performance**, n (%) | |  | |  | 0.2178^1^ | |  | |  | 0.0731^1^ | |  |  | | 0.0361^1^ |
| Agree | | 38 (84.4%) | | 9 (69.2%) |  | | 37 (86.0%) | | 9 (64.3%) |  | | 25 (92.6%) | 22 (71.0%) | |  |
| Do Not Agree | | 7 (15.6%) | | 4 (30.8%) |  | | 6 (14.0%) | | 5 (35.7%) |  | | 2 (7.4%) | 9 (29.0%) | |  |
| **Develops a trusting relationship**, n (%) | |  | |  | 0.1427^1^ | |  | |  | 0.0002^1^ | |  |  | | 0.0643^1^ |
| Agree | | 39 (86.7%) | | 9 (69.2%) |  | | 40 (93.0%) | | 7 (50.0%) |  | | 25 (92.6%) | 23 (74.2%) | |  |
| Do Not Agree | | 6 (13.3%) | | 4 (30.8%) |  | | 3 (7.0%) | | 7 (50.0%) |  | | 2 (7.4%) | 8 (25.8%) | |  |
| **Is accommodating to different communication styles**, n (%) | |  | |  | 0.0659^1^ | |  | |  | 0.0005^1^ | |  |  | | 0.0310^1^ |
| Agree | | 33 (73.3%) | | 6 (46.2%) |  | | 34 (79.1%) | | 4 (28.6%) |  | | 22 (81.5%) | 17 (54.8%) | |  |
| Do Not Agree | | 12 (26.7%) | | 7 (53.8%) |  | | 9 (20.9%) | | 10 (71.4%) |  | | 5 (18.5%) | 14 (45.2%) | |  |
| **Persues strategies to improve communication**, n (%) | |  | |  | 0.0954^1^ | |  | |  | 0.0010^1^ | |  |  | | 0.0170^1^ |
| Agree | | 32 (71.1%) | | 6 (46.2%) |  | | 33 (76.7%) | | 4 (28.6%) |  | | 22 (81.5%) | 16 (51.6%) | |  |
| Do Not Agree | | 13 (28.9%) | | 7 (53.8%) |  | | 10 (23.3%) | | 10 (71.4%) |  | | 5 (18.5%) | 15 (48.4%) | |  |
| **Coordinates and collaborates with other mentors**, n (%) | |  | |  | 0.0446^1^ | |  | |  | 0.1063^1^ | |  |  | | 0.2655^1^ |
| Agree | | 28 (62.2%) | | 4 (30.8%) |  | | 26 (60.5%) | | 5 (35.7%) |  | | 17 (63.0%) | 15 (48.4%) | |  |
| Do Not Agree | | 17 (37.8%) | | 9 (69.2%) |  | | 17 (39.5%) | | 9 (64.3%) |  | | 10 (37.0%) | 16 (51.6%) | |  |
| **Sets clear mentorship expectations**, n (%) | |  | |  | 0.1690^1^ | |  | |  | 0.0256^1^ | |  |  | | 0.0299^1^ |
| Agree | | 27 (60.0%) | | 5 (38.5%) |  | | 27 (62.8%) | | 4 (28.6%) |  | | 19 (70.4%) | 13 (41.9%) | |  |
| Do Not Agree | | 18 (40.0%) | | 8 (61.5%) |  | | 16 (37.2%) | | 10 (71.4%) |  | | 8 (29.6%) | 18 (58.1%) | |  |
| **Aligns with your mentorship expectations**, n (%) | |  | |  | 0.0659^1^ | |  | |  | 0.0005^1^ | |  |  | | <.0001^1^ |
| Agree | | 33 (73.3%) | | 6 (46.2%) |  | | 34 (79.1%) | | 4 (28.6%) |  | | 26 (96.3%) | 13 (41.9%) | |  |
| Do Not Agree | | 12 (26.7%) | | 7 (53.8%) |  | | 9 (20.9%) | | 10 (71.4%) |  | | 1 (3.7%) | 18 (58.1%) | |  |
| **Is considerate about the mentor-mentee differences**, n (%) | |  | |  | 0.1330^1^ | |  | |  | 0.0143^1^ | |  |  | | 0.0386^1^ |
| Agree | | 31 (68.9%) | | 6 (46.2%) |  | | 31 (72.1%) | | 5 (35.7%) |  | | 21 (77.8%) | 16 (51.6%) | |  |
| Do Not Agree | | 14 (31.1%) | | 7 (53.8%) |  | | 12 (27.9%) | | 9 (64.3%) |  | | 6 (22.2%) | 15 (48.4%) | |  |
| **Sets clear goals**, n (%) | |  | |  | 0.3001^1^ | |  | |  | 0.0015^1^ | |  |  | | 0.0257^1^ |
| Agree | | 28 (62.2%) | | 6 (46.2%) |  | | 30 (69.8%) | | 3 (21.4%) |  | | 20 (74.1%) | 14 (45.2%) | |  |
| Do Not Agree | | 17 (37.8%) | | 7 (53.8%) |  | | 13 (30.2%) | | 11 (78.6%) |  | | 7 (25.9%) | 17 (54.8%) | |  |
| **Helps me develop strategies to meet my goals**, n (%) | |  | |  | 0.0038^1^ | |  | |  | <.0001^1^ | |  |  | | 0.0039^1^ |
| Agree | | 30 (68.2%) | | 3 (23.1%) |  | | 31 (72.1%) | | 1 (7.7%) |  | | 21 (77.8%) | 12 (40.0%) | |  |
| Do Not Agree | | 14 (31.8%) | | 10 (76.9%) |  | | 12 (27.9%) | | 12 (92.3%) |  | | 6 (22.2%) | 18 (60.0%) | |  |
| **Estimates my knowledge, abilities and skills**, n (%) | |  | |  | 0.5111^1^ | |  | |  | 0.0024^1^ | |  |  | | 0.1758^1^ |
| Agree | | 32 (71.1%) | | 8 (61.5%) |  | | 34 (79.1%) | | 5 (35.7%) |  | | 21 (77.8%) | 19 (61.3%) | |  |
| Do Not Agree | | 13 (28.9%) | | 5 (38.5%) |  | | 9 (20.9%) | | 9 (64.3%) |  | | 6 (22.2%) | 12 (38.7%) | |  |
| **Enhances my knowledge, abilities and skills**, n (%) | |  | |  | 0.1257^1^ | |  | |  | 0.0005^1^ | |  |  | | 0.0086^1^ |
| Agree | | 36 (81.8%) | | 8 (61.5%) |  | | 37 (88.1%) | | 6 (42.9%) |  | | 25 (92.6%) | 19 (63.3%) | |  |
| Do Not Agree | | 8 (18.2%) | | 5 (38.5%) |  | | 5 (11.9%) | | 8 (57.1%) |  | | 2 (7.4%) | 11 (36.7%) | |  |
| **Inspires me and motivates me to meet my goals, and maximize my potential**, n (%) | |  | |  | 0.1706^1^ | |  | |  | <.0001^1^ | |  |  | | 0.0007^1^ |
| Agree | | 36 (80.0%) | | 8 (61.5%) |  | | 39 (90.7%) | | 4 (28.6%) |  | | 26 (96.3%) | 18 (58.1%) | |  |
| Do Not Agree | | 9 (20.0%) | | 5 (38.5%) |  | | 4 (9.3%) | | 10 (71.4%) |  | | 1 (3.7%) | 13 (41.9%) | |  |
| **Empowers me to do my job**, n (%) | |  | |  | 0.3192^1^ | |  | |  | 0.0005^1^ | |  |  | | 0.0423^1^ |
| Agree | | 34 (75.6%) | | 8 (61.5%) |  | | 36 (83.7%) | | 5 (35.7%) |  | | 23 (85.2%) | 19 (61.3%) | |  |
| Do Not Agree | | 11 (24.4%) | | 5 (38.5%) |  | | 7 (16.3%) | | 9 (64.3%) |  | | 4 (14.8%) | 12 (38.7%) | |  |
| **Stimulates my creative thinking**, n (%) | |  | |  | 0.0119^1^ | |  | |  | 0.0001^1^ | |  |  | | 0.0729^1^ |
| Agree | | 35 (81.4%) | | 6 (46.2%) |  | | 36 (85.7%) | | 4 (30.8%) |  | | 22 (84.6%) | 19 (63.3%) | |  |
| Do Not Agree | | 8 (18.6%) | | 7 (53.8%) |  | | 6 (14.3%) | | 9 (69.2%) |  | | 4 (15.4%) | 11 (36.7%) | |  |
| **Acknowledges my professional contributions and gives me appropriate credit**, n (%) | |  | |  | 0.0020^1^ | |  | |  | 0.0052^1^ | |  |  | | 0.0541^1^ |
| Agree | | 39 (86.7%) | | 6 (46.2%) |  | | 37 (86.0%) | | 7 (50.0%) |  | | 24 (88.9%) | 21 (67.7%) | |  |
| Do Not Agree | | 6 (13.3%) | | 7 (53.8%) |  | | 6 (14.0%) | | 7 (50.0%) |  | | 3 (11.1%) | 10 (32.3%) | |  |
| **Fosters my independence**, n (%) | |  | |  | 0.0045^1^ | |  | |  | 0.0011^1^ | |  |  | | 0.0305^1^ |
| Agree | | 38 (84.4%) | | 6 (46.2%) |  | | 37 (86.0%) | | 6 (42.9%) |  | | 24 (88.9%) | 20 (64.5%) | |  |
| Do Not Agree | | 7 (15.6%) | | 7 (53.8%) |  | | 6 (14.0%) | | 8 (57.1%) |  | | 3 (11.1%) | 11 (35.5%) | |  |
| **Accounts for differences in my gender and cultural background**, n (%) | |  | |  | 0.1654^1^ | |  | |  | 0.1287^1^ | |  |  | | 0.2172^1^ |
| Agree | | 20 (44.4%) | | 3 (23.1%) |  | | 19 (44.2%) | | 3 (21.4%) |  | | 13 (48.1%) | 10 (32.3%) | |  |
| Do Not Agree | | 25 (55.6%) | | 10 (76.9%) |  | | 24 (55.8%) | | 11 (78.6%) |  | | 14 (51.9%) | 21 (67.7%) | |  |
| **Helps me network effectively**, n (%) | |  | |  | 0.0206^1^ | |  | |  | 0.0105^1^ | |  |  | | 0.2456^1^ |
| Agree | | 30 (66.7%) | | 4 (30.8%) |  | | 29 (67.4%) | | 4 (28.6%) |  | | 18 (66.7%) | 16 (51.6%) | |  |
| Do Not Agree | | 15 (33.3%) | | 9 (69.2%) |  | | 14 (32.6%) | | 10 (71.4%) |  | | 9 (33.3%) | 15 (48.4%) | |  |
| **Helps me develop a personalized career path that aligns with my goals**, n (%) | |  | |  | 0.0098^1^ | |  | |  | 0.0116^1^ | |  |  | | 0.0275^1^ |
| Agree | | 31 (70.5%) | | 4 (30.8%) |  | | 30 (69.8%) | | 4 (30.8%) |  | | 20 (76.9%) | 15 (48.4%) | |  |
| Do Not Agree | | 13 (29.5%) | | 9 (69.2%) |  | | 13 (30.2%) | | 9 (69.2%) |  | | 6 (23.1%) | 16 (51.6%) | |  |
| **Helps me achieve an adequate work/life balance**, n (%) | |  | |  | 0.3080^1^ | |  | |  | 0.0152^1^ | |  |  | | 0.0204^1^ |
| Agree | | 21 (46.7%) | | 4 (30.8%) |  | | 22 (51.2%) | | 2 (14.3%) |  | | 16 (59.3%) | 9 (29.0%) | |  |
| Do Not Agree | | 24 (53.3%) | | 9 (69.2%) |  | | 21 (48.8%) | | 12 (85.7%) |  | | 11 (40.7%) | 22 (71.0%) | |  |
| **Is my role model**, n (%) | |  | |  | 0.8058^1^ | |  | |  | 0.0045^1^ | |  |  | | 0.0275^1^ |
| Agree | | 28 (62.2%) | | 7 (58.3%) |  | | 30 (71.4%) | | 4 (28.6%) |  | | 20 (76.9%) | 15 (48.4%) | |  |
| Do Not Agree | | 17 (37.8%) | | 5 (41.7%) |  | | 12 (28.6%) | | 10 (71.4%) |  | | 6 (23.1%) | 16 (51.6%) | |  |
| **Has professional and mentoring experience that facilitates my development**, n (%) | |  | |  | 0.1891^1^ | |  | |  | 0.0015^1^ | |  |  | | 0.0165^1^ |
| Agree | | 34 (77.3%) | | 7 (58.3%) |  | | 35 (83.3%) | | 5 (38.5%) |  | | 23 (88.5%) | 18 (60.0%) | |  |
| Do Not Agree | | 10 (22.7%) | | 5 (41.7%) |  | | 7 (16.7%) | | 8 (61.5%) |  | | 3 (11.5%) | 12 (40.0%) | |  |
| **Facilitates access to research and professional resources**, n (%) | |  | |  | 0.1810^1^ | |  | |  | 0.0178^1^ | |  |  | | 0.1758^1^ |
| Agree | | 33 (73.3%) | | 7 (53.8%) |  | | 33 (76.7%) | | 6 (42.9%) |  | | 21 (77.8%) | 19 (61.3%) | |  |
| Do Not Agree | | 12 (26.7%) | | 6 (46.2%) |  | | 10 (23.3%) | | 8 (57.1%) |  | | 6 (22.2%) | 12 (38.7%) | |  |
| ^1^Chi-Square p-value; |  | |  | | |  | |  | | |  | | |  | |

| **Variable** | **Model 1:** *Including the interaction term for Mentor inspires and motivates me * Region* | | | **Model 2:** *Including the interaction term for Mentor provides helpful feedback * Region* | | | **Model 3:** *including the interaction term for Mentor helps develop career path * Region.* | | |
| --- | --- | --- | --- | --- | --- | --- | --- | --- | --- |
|  | OR | 95% CI | p – value | OR | 95% CI | p – value | OR | 95% CI | p – value |
| Number of years in training after completion of medical school (Per 1 year increase) | 0.99 | 0.695 - 1.433 | 0.979 | 0.95 | 0.660 - 1.386 | 0.798 | 0.96 | 0.661 - 1.410 | 0.837 |
| Sex (Men vs. Women) | 0.81 | 0.311 - 2.184 | 0.675 | 0.79 | 0.307 - 2.109 | 0.634 | 0.77 | 0.301 - 2.067 | 0.606 |
| Region (Europe vs. US) | 3.14 | 0.655 - 16.900 | 0.161 | 1.69 | 0.276 - 11.181 | 0.570 | 0.93 | 0.221 - 3.930 | 0.930 |
| Mentor helps me develop personalized career path  (Agree vs Disagree) | 2.96 | 0.908 - 9.414 | 0.066 | 3.28 | 1.019 - 10.424 | 0.043 | 3.75 | 0.822 - 16.574 | 0.081 |
| Mentor provides helpful feedback  (Agree vs Disagree) | 0.76 | 0.150 - 3.317 | 0.730 | 1.21 | 0.195 - 7.049 | 0.826 | 0.79 | 0.166 - 3.401 | 0.768 |
| Mentor inspires and motivates me  (Agree vs Disagree) | 14.90 | 2.598 - 101.962 | 0.003 | 5.04 | 1.280 - 21.196 | 0.022 | 4.94 | 1.249 - 21.153 | 0.025 |
| Mentor inspires and motivates me * Region (Europe *Agree) | 0.12 | 0.017 - 0.914 | 0.042 | - | - | - | - | - | - |
| Mentor provides helpful feedback * Region (Europe *Agree) | - | - | - | 0.40 | 0.049 - 3.336 | 0.402 | - | - | - |
| Mentor helps develop career path * Region (Europe *Agree) | - | - | - | - | - | - | 0.90 | 0.137 - 6.547 | 0.915 |

**eTable 5a. Multivariable Models of Academic Productivity, Assessing the Interaction Between Mentorship Factors and Geographic Region**

US: United States; OR: Odds ratio; CI: Confidence interval

**eTable 5b. Multivariable Models of Burnout, Assessing the Interaction Between Mentorship Factors and Geographic Region**

| **Variable** | **Model 1:** *Including the interaction term for Mentor helps me achieve work/life balance * Region* | | | **Model 2:** *Including the interaction term for Mentor helps develop strategies to meet goals * Region* | | | **Model 3:** *including the interaction term for* Mentor empowers me to do my job * Region | | |
| --- | --- | --- | --- | --- | --- | --- | --- | --- | --- |
|  | OR | 95% CI | p – value | OR | 95% CI | p – value | OR | 95% CI | p – value |
| Number of years in training after completion of medical school (Per 1 year increase) | 1.65 | 1.184 - 2.409 | 0.005 | 1.59 | 1.151 - 2.276 | 0.007 | 1.60 | 1.163 - 2.291 | 0.006 |
| Sex (Men vs. Women) | 1.06 | 0.497 - 2.307 | 0.879 | 1.08 | 0.508 - 2.354 | 0.837 | 1.08 | 0.508 - 2.356 | 0.837 |
| Region (Europe vs. US) | 0.36 | 0.060 - 1.944 | 0.243 | 0.83 | 0.222 - 3.107 | 0.783 | 0.68 | 0.101 - 4.279 | 0.685 |
| Mentor helps me achieve work/life balance  (Agree vs Disagree) | 1.78 | 0.428 - 7.791 | 0.425 | 2.70 | 0.873 - 8.689 | 0.086 | 2.68 | 0.866 - 8.658 | 0.089 |
| Mentor helps develop strategies to meet goals  (Agree vs Disagree) | 4.17 | 1.684 - 10.807 | 0.002 | 4.51 | 1.509 - 14.447 | 0.008 | 4.08 | 1.655 - 10.515 | 0.003 |
| Mentor empowers me to do my job (Agree vs Disagree) | 2.15 | 0.626 - 7.708 | 0.223 | 1.98 | 0.585 - 6.923 | 0.270 | 1.98 | 0.451 - 9.406 | 0.368 |
| Mentor helps me achieve work/life balance * Region (Europe *Agree) | - | - | - | 0.76 | 0.152 - 4.013 | 0.751 | - | - | - |
| Mentor helps develop strategies to meet goals * Region (Europe *Agree) | 2.35 | 0.366 - 16.769 | 0.373 | - | - | - | - | - | - |
| Mentor empowers me to do my job * Region (Europe *Agree) | - | - | - | - | - | - | 1.04 | 0.144 - 8.130 | 0.368 |

US: United States; OR: Odds ratio; CI: Confidence interval

**eTable 5c. Multivariable Models of Mentorship satisfaction, Assessing the Interaction Between Mentorship Factors and Geographic Region**

| **Variable** | **Model 1:** *Including the interaction term for Aligns with mentorship expectations * Region* | | | **Model 2:** *Including the interaction term for Mentor inspires and motivates me * Region* | | | **Model 3:** *Including the interaction term for Mentor acknowledges my contributions and gives me credit * Region* | | |
| --- | --- | --- | --- | --- | --- | --- | --- | --- | --- |
|  | OR | 95% CI | p – value | OR | 95% CI | p – value | OR | 95% CI | p – value |
| Number of years in training after completion of medical school (Per 1 year increase) | 0.65 | 0.405 - 1.014 | 0.070 | 0.58 | 0.324 - 0.949 | 0.045 | 0.62 | 0.373 - 0.981 | 0.052 |
| Sex (Men vs. Women) | 1.22 | 0.374 - 4.234 | 0.743 | 1.13 | 0.343 - 4.029 | 0.838 | 1.18 | 0.362 - 4.169 | 0.781 |
| Region (Europe vs. US) | 0.91 | 0.180 - 4.502 | 0.908 | 0.38 | 0.057 - 2.218 | 0.300 | 0.49 | 0.062 - 3.470 | 0.482 |
| Aligns with mentorship expectations (Agree vs Disagree) | 7.17 | 1.090 - 50.981 | 0.040 | 5.91 | 1.342 - 26.514 | 0.018 | 5.28 | 1.201 - 23.163 | 0.025 |
| Mentor inspires and motivates me (Agree vs Disagree) | 4.32 | 3.235 - 75.866 | <0.001 | 8.26 | 1.223 - 62.335 | 0.033 | 17.07 | 3.695, 95.839 | <0.001 |
| Mentor acknowledges my contributions and gives me credit (Agree vs Disagree) | 0.65 | 0.113 - 3.062 | 0.604 | 0.69 | 0.118 - 3.475 | 0.670 | 0.44 | 0.048 - 3.295 | 0.450 |
| Aligns with mentorship expectations * Region (Europe *Agree) | 0.59 | 0.056 - 6.430 | 0.658 | - | - | - | - | - | - |
| Mentor inspires and motivates me * Region (Europe *Agree) | **-** | - | - | 3.70 | 0.303 - 62.931 | 0.328 | - | - | - |
| Mentor acknowledges my contributions and gives me credit * Region (Europe *Agree) | - | - | - | - | - | - | 1.89 | 0.165 - 26.557 | 0.617 |

US: United States; OR: Odds ratio; CI: Confidence interval
